# Unveiling the Hidden Power of Uromodulin: A Promising Potential Biomarker for Kidney Diseases

**DOI:** 10.3390/diagnostics13193077

**Published:** 2023-09-28

**Authors:** Raïsa Thielemans, Reinhart Speeckaert, Charlotte Delrue, Sander De Bruyne, Matthijs Oyaert, Marijn M. Speeckaert

**Affiliations:** 1 Department of Nephrology, Ghent University Hospital, 9000 Ghent, Belgium; raisa.thielemans@ugent.be (R.T.); charlotte.delrue@ugent.be (C.D.); 2 Department of Dermatology, Ghent University Hospital, 9000 Ghent, Belgium; reinhart.speeckaert@uzgent.be; 3 Department of Laboratory Medicine, Ghent University Hospital, 9000 Ghent, Belgium; sander.debruyne@uzgent.be (S.D.B.); matthijs.oyaert@uzgent.be (M.O.); 4 Research Foundation Flanders, 1000 Brussels, Belgium

**Keywords:** uromodulin, acute kidney injury, chronic kidney disease, Tamm-Horsfall protein

## Abstract

Uromodulin, also known as Tamm-Horsfall protein, represents the predominant urinary protein in healthy individuals. Over the years, studies have revealed compelling associations between urinary and serum concentrations of uromodulin and various parameters, encompassing kidney function, graft survival, cardiovascular disease, glucose metabolism, and overall mortality. Consequently, there has been a growing interest in uromodulin as a novel and effective biomarker with potential applications in diverse clinical settings. Reduced urinary uromodulin levels have been linked to an elevated risk of acute kidney injury (AKI) following cardiac surgery. In the context of chronic kidney disease (CKD) of different etiologies, urinary uromodulin levels tend to decrease significantly and are strongly correlated with variations in estimated glomerular filtration rate. The presence of uromodulin in the serum, attributable to basolateral epithelial cell leakage in the thick ascending limb, has been observed. This serum uromodulin level is closely associated with kidney function and histological severity, suggesting its potential as a biomarker capable of reflecting disease severity across a spectrum of kidney disorders. The *UMOD* gene has emerged as a prominent locus linked to kidney function parameters and CKD risk within the general population. Extensive research in multiple disciplines has underscored the biological significance of the top *UMOD* gene variants, which have also been associated with hypertension and kidney stones, thus highlighting the diverse and significant impact of uromodulin on kidney-related conditions. *UMOD* gene mutations are implicated in uromodulin-associated kidney disease, while polymorphisms in the *UMOD* gene show a significant association with CKD. In conclusion, uromodulin holds great promise as an informative biomarker, providing valuable insights into kidney function and disease progression in various clinical scenarios. The identification of *UMOD* gene variants further strengthens its relevance as a potential target for better understanding kidney-related pathologies and devising novel therapeutic strategies. Future investigations into the roles of uromodulin and regulatory mechanisms are likely to yield even more profound implications for kidney disease diagnosis, risk assessment, and management.

## 1. Introduction

In 1873, an Italian physician named Carlo Rovida (University of Turin, Turin, Italy) discovered a substance produced by tubular cells in the kidney. He observed that this substance, referred to as cilindrina, led to the formation of hyaline casts in the tubular lumen [1]. The Tamm-Horsfall protein, discovered by Tamm and Horsfall in 1950, is the predominant protein excreted in urine by epithelial cells lining the thick ascending limb (TAL) of the loop of Henle (90%) and by epithelial cells of the early part of the distal convoluted tubule (DCT1) (10%) [2,3]. Uromodulin primarily localizes to the apical membrane within TAL epithelial cells, although it has also been observed at the basolateral membrane, which is important for the release of this protein in the renal interstitium and subsequently into the systemic circulation [4,5]. Uromodulin expression gradually increases as the TAL segments mature, ultimately reaching its peak level after birth. Uromodulin excretion demonstrates a gradual increase from birth to adulthood [6], stabilizes during the adult years [7,8,9,10], and subsequently starts to decline after the age of 60 years [11,12]. Moreover, it has been documented that this protein can interact with and impede viral hemagglutination [2,3]. Muchmore and Decker made significant discoveries in 1985. They found that an 85 kDa glycoprotein extracted from the urine of pregnant women possessed the remarkable capability to inhibit antigen-induced T-cell proliferation and monocyte cytotoxicity when tested in vitro, for which it was named uromodulin [13]. Subsequently, Pennica et al. determined through sequence analysis that Tamm-Horsfall protein and uromodulin are identical proteins [14]. Uromodulin is only detected in the kidneys, excreted into both the urine and interstitium, and circulates in the bloodstream [4,15,16]. Water loading leads to an increase in the secretion of polymerizing urinary uromodulin. This heightened secretion contributes to a decrease in the variability of urinary uromodulin concentrations, even when dealing with high urine volumes [17]. In various forms of kidney disease, interstitial uromodulin is closely correlated with serum uromodulin concentrations, although there is a concentration gradient between the medulla and cortex of the kidney [4].

The *UMOD* gene, located on chromosome 16p12.3–16p13.11, is composed of 11 exons (the first of which is noncoding) over a genomic region of about 20 kb and encodes for uromodulin [14,18]. Among the multitude of genetic loci associated with chronic kidney disease (CKD) in the general population, the *UMOD* locus is particularly significant and exerts a substantial impact on both the estimated glomerular filtration rate (eGFR) and CKD risk. Notably, this effect remains consistent across diverse ethnic groups and is associated with age [19]. In addition, rare *UMOD* mutations have been identified in patients diagnosed with familial juvenile hyperuricemic nephropathy (FJHN), medullary cystic kidney disease 2 (MCKD2), and autosomal dominant tubulointerstitial kidney disease (ADTKD) [20,21]. In addition, genome-wide association studies (GWAS) have demonstrated that common variants located in the promoter region of the *UMOD* gene are linked to CKD, GFR, kidney stone formation, and arterial hypertension, and increase *UMOD* expression and excretion of uromodulin in urine [22,23,24,25]. In the present review, we will give an overview of the general characteristics of uromodulin and its role in a wide spectrum of kidney diseases.

## 2. Methodology

This narrative review was based on a literature search in the online PubMed Database, which was conducted by the authors to identify relevant studies published between 2000 and 2022. The search terms included “uromodulin”, “kidney disease”, “biomarker”, “glomerular filtration rate”, “chronic kidney disease”, and “acute kidney injury”. The authors also searched the reference lists of the relevant articles to identify additional studies. The inclusion criteria for the studies were as follows: (1) original research articles published in peer-reviewed journals; (2) studies that investigated the association between uromodulin and kidney function, kidney disease, or other clinical outcomes; and (3) studies that reported quantitative data on uromodulin levels. The exclusion criteria were studies that were not written in English and did not have an abstract in English were excluded. The authors extracted data from the included studies, including the study design, sample size, patient characteristics, uromodulin measurement methods, and outcomes.

## 3. Uromodulin: General Characteristics

### 3.1. Synthesis of Uromodulin

**Figure 1:**
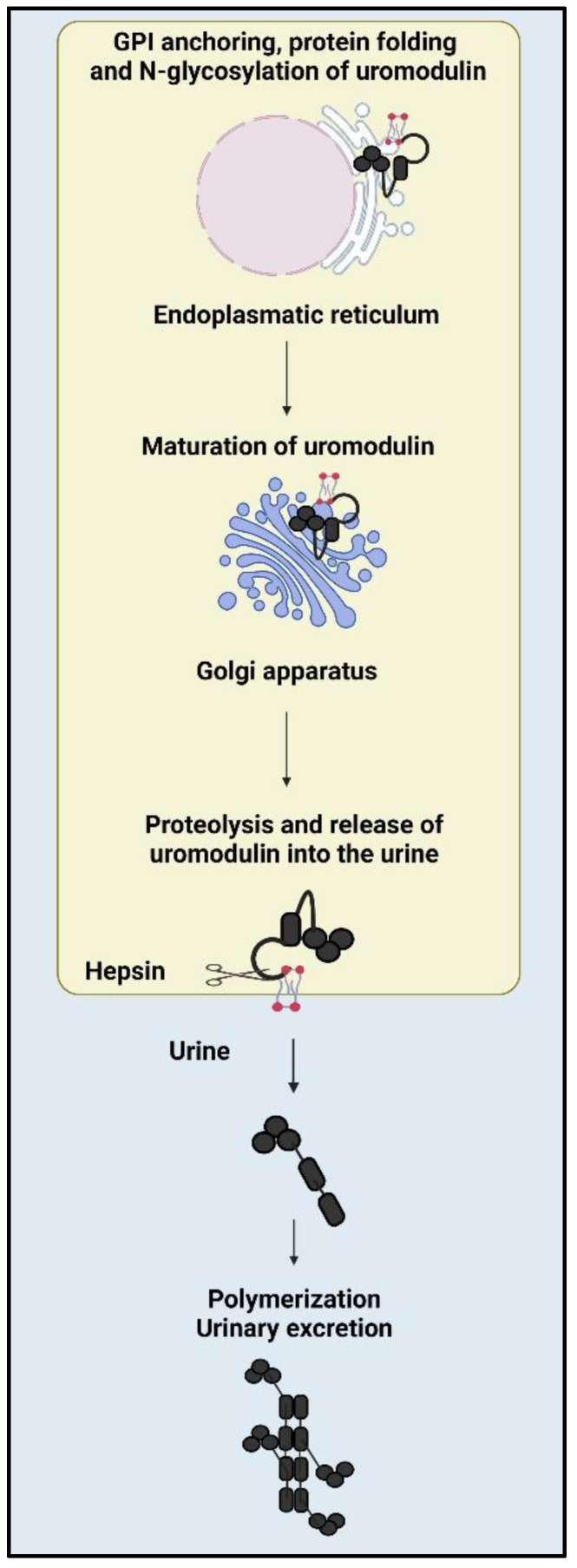
Phases of uromodulin processing and excretion in kidney epithelial cells. Uromodulin, a protein produced by kidney epithelial cells, undergoes a complex maturation and excretion process, contributing to its functional roles in the urinary tract. Newly synthesized uromodulin undergoes glycosylphosphatidylinositol (GPI) anchoring in the endoplasmic reticulum, which attaches a GPI lipid anchor to its C-terminus, enabling its association with the endoplasmatic reticulum membrane. Proper protein folding occurs, ensuring the acquisition of its functional three-dimensional conformation. Furthermore, N-glycosylation takes place, involving the addition of oligosaccharide chains to specific asparagine residues, which enhances its stability and intracellular trafficking. Uromodulin is transported from the endoplasmic reticulum to the Golgi apparatus, where it undergoes further maturation. Sorting into vesicles occurs within the Golgi complex. Post-translational modifications continue, including the trimming and processing of N-glycans and refining uromodulin’s glycosylation pattern. This Golgi maturation process is essential for quality control and prepares uromodulin for transport to the cell surface. At the apical surface of kidney epithelial cells, mature uromodulin becomes incorporated into forming urinary casts. Here, it undergoes proteolytic processing mediated by the serine protease hepsin. This cleavage produces bioactive fragments of uromodulin, which are subsequently released into the urine along with the urinary casts during excretion.

Uromodulin is initially synthesized as a 640-amino acid precursor. The protein possesses a leader peptide (24 N-terminal amino acids) that facilitates its co-translational insertion into the endoplasmic reticulum, where it undergoes cleavage during maturation (Figure 1). Additionally, a hydrophobic segment is present at the C-terminal (615–640), functioning as a signal for the attachment of a glycosylphosphatidylinositol (GPI) membrane anchor at position 614 [26]. As a result of this GPI anchor, uromodulin is a well-organized, detergent-insoluble glycosphingolipid complex, also known as lipid rafts, which are enriched in cholesterol and sphingolipids. These microdomains play a crucial role in organizing the trafficking of specific membranes and associated proteins, thereby acting as platforms for intracellular signaling [27]. The mature urinary protein consists of 616 amino acids with a deduced molecular weight of 67 kDa [14,28,29,30]. Uromodulin exhibits a substantial carbohydrate content of 30% (linked to its immunosuppressive properties) [31], a notable abundance of cysteine residues, with 48 present in the mature protein (essential for correct protein folding), and a predisposition to form substantial aggregates [32]. In human uromodulin, eight potential Asn N-linked glycosylation sites have been identified (Asn38, Asn76, Asn80, Asn232, Asn275, Asn322, Asn396, and Asn513), with all except Asn38 being glycosylated [33]. The maturation process of the protein along the secretory pathway is relatively slow, with approximately 8–12 h in stably transfected HeLa cells. This process involves membrane anchoring through GPI and the involvement of all 48 cysteine residues to form 24 intramolecular disulfide bonds within the oxidizing environment of the endoplasmic reticulum [14,31,34]. During the maturation of uromodulin in the Golgi, high-mannose residues are converted to complex glycans, primarily of the polyantennary type, which can undergo sialylation, fucosylation, or sulfation. This maturation process results in an apparent increase in molecular mass from ~85 kDa to 100 kDa under reducing conditions. One N-glycosylation site (Asn275) remains unprocessed by the Golgi and retains its high-mannose chain [33,35]. Due to the presence of sialic acid residues, the isoelectric point of uromodulin is approximately 3.5, making it a polyanionic protein in urine [36]. 

Notably, uromodulin features a unique arrangement of terminal sugars, forming the Sda antigen, a dominant blood group determinant found in 90% of the white population [37,38]. Furthermore, there is evidence suggesting O-glycosylation in uromodulin, which may be regulated by hormones [39]. Although several conserved phosphorylation sites have been identified, their physiological significance remains to be fully understood [14,28,29,30]. 

### 3.2. Composition of Uromodulin

Uromodulin comprises several distinct domains, namely four epidermal growth factor (EGF)-like domains, with EGF-II and EGF-III likely acting as Ca^2+^ binding sites [40]. Additionally, there is a cysteine-rich domain of uncertain function (D8C) along with a bipartite elastase-resistant C-terminal zona pellucida (ZP) domain [41]. EGF-like domains are known for their significance in protein-protein interactions [42], whereas ZP domains play a vital role in assembling these proteins into supramolecular extracellular polymers with a high structural organization [41]. During intracellular trafficking, uromodulin undergoes hydrophobic interactions involving two motifs, namely, the internal (IHP) and external hydrophobic patches (EHP). These motifs are situated in the ZP linker region and between the proteolytic cleavage and GPI-anchoring sites. This intramolecular interaction is essential for accurate proteolytic cleavage of the apical membrane [43]. Uromodulin, when targeted apically, is released into the urine through proteolytic cleavage at a conserved site (Phe587) located just at the C-terminal of the ZP domain [44]. This proteolytic cleavage is facilitated by the type II transmembrane serine protease hepsin (Figure 1), which releases external hydrophobic patches at the COOH-terminus, thus enabling proper polymerization and subsequent release of the protein into the urine. In mice lacking hepsin, uromodulin secretion is significantly reduced, leading to protein accumulation in the kidneys. Furthermore, urinary uromodulin in these mice retains EHPs, preventing polymerization and rendering it non-polymeric [41,43,45]. Unlike urinary uromodulin, circulating uromodulin does not form aggregates. An alternative processing pathway that preserves the EHP domain can result in the non-polymerizing form of uromodulin [46]. Urinary uromodulin exists as a high-molecular-weight polymer, approximately 7 × 10^6^ Da, or multiples thereof, contributing to its distinctive properties [47]. Under electron microscopy, the protein adopts a porous, three-dimensional matrix structure that is easily discernible. The matrix structure shaped by uromodulin displays elongated protein filaments comprising smaller fibrils measuring approximately 100 Å in width and having an average length of 25,000 Å [48]. Each uromodulin filament consists of two protofilaments arranged in a right-handed double helix, exhibiting an axial repeat of approximately 120 Å and a diameter ranging from 90 to 140 Å [41]. The precise structural arrangement of uromodulin filaments is influenced by ionic conditions, with maximum compaction observed when NaCl and CaCl_2_ concentrations approach those present in the pro-urine facing the TAL [49]. 

### 3.3. Production and Secretion of Uromodulin

Uromodulin is the predominant protein in healthy urine, boasting mean daily secretion rates of 0.3 mg per 100 g body weight in rats and ranging from 50 to 150 mg per 24 h in humans [47,48,50,51]. Additionally, a fraction of the uromodulin produced in the TAL is released into the bloodstream [4]. Plasma uromodulin concentrations are approximately 1000 times lower than urinary levels [52,53]. The estimated half-life of human uromodulin is approximately 16 h [54]. Urinary excretion of uromodulin is characterized by significant fluctuations that occur both within and among individuals. Moreover, considerable unexplained variation in urinary uromodulin levels has been observed across different isolated and non-isolated populations [54,55]. Our understanding of the physiological regulators influencing uromodulin abundance in the kidney and urine remains limited. Various indications point to the salt sensitivity of urinary uromodulin excretion, showing a positive correlation with salt intake in humans [22,56]. Uromodulin expression may also be influenced by various factors, such as protein intake [50], antidiuretic hormone [50,57], and thyroid hormones [58]. Additional research is required to elucidate the mechanisms governing uromodulin production in TAL cells and its subsequent release into the urine. 

### 3.4. Functions

**Figure 2:**
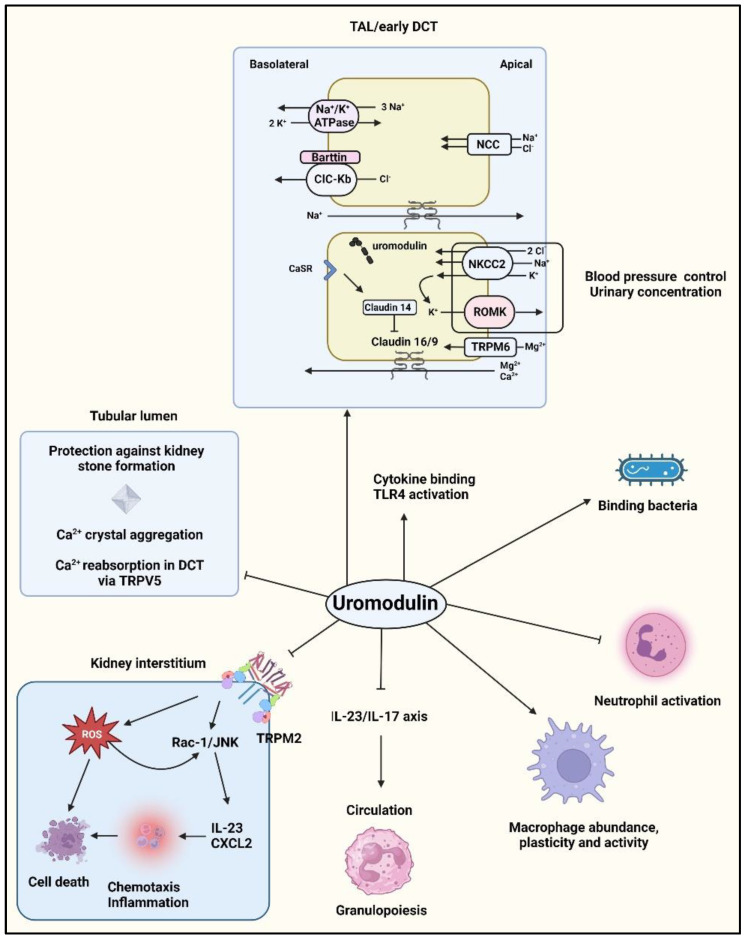
Uromodulin, a protein predominantly produced by kidney epithelial cells, plays a diverse role in human physiology. It regulates the renal outer medullary potassium channel (ROMK) and sodium-potassium-chloride transporter (NKCC2) in the thick ascending limb of the loop of Henle, impacting potassium and electrolyte balance. In the early distal convoluted tubule, it modulates the Na^+^/Cl^−^ cotransporter (NCC) to regulate sodium and water reabsorption. Uromodulin also influences calcium homeostasis through interactions with transient receptor potential cation channels subfamily V members 5 and 6 (TRPV5/6) and magnesium homeostasis via transient receptor potential melastatin 6 (TRPM6). Moreover, it plays a role in blood pressure control, promotes urinary cast formation, protects against kidney stone formation by inhibiting calcium crystal aggregation, and exhibits antimicrobial properties, inhibiting urinary tract infections.

Despite its widespread use, the precise function of uromodulin remains unclear [59]. Uromodulin exhibits diverse physiological functions (Figure 2), including regulation of the renal outer medullary potassium channel (ROMK), sodium-potassium-chloride transporter (NKCC2) activity [60,61], Na^+^/Cl^−^ cotransporter (NCC) in the early distal convoluted tubule [62], calcium (via transient receptor potential cation channel subfamily V members 5 and 6, TRPV5/6) [63] and magnesium homeostasis through the ion channel transient receptor potential melastatin 6 (TRPM6) [63,64], blood pressure control [65], promotion of urinary cast formation [38,66], protection against kidney stones by reducing the aggregation of calcium crystals, and inhibition of urinary tract infections [67]. Uromodulin interacts with type 1 fimbriae of *E. coli*, effectively obstructing urothelial cell colonization [68]. A comprehensive investigation involving a cohort of 953 community-dwelling elderly subjects enrolled in the Cardiovascular Health Study revealed that individuals exhibiting elevated urinary uromodulin levels at baseline experienced a reduced risk of urinary tract infections [69]. These findings align with the results of a case-control study that compared controls with urinary tract infection patients with or without bacteremic *E. coli*. In this study, it was observed that subjects with exceptionally low uromodulin levels were more prevalent in the group of patients with bacteremia [70]. Uromodulin plays a vital role in the urinary innate immune defense, making its biochemical and structural characteristics potential targets for leveraging or adjusting its excretion rates to prevent or treat urinary tract infections [59]. Research in mice [71,72,73,74,75] and genetic studies [76] have suggested that uromodulin inhibits the colonization of urothelial cells by bacteria. Uromodulin forms filament structures that hinder uropathogen attachment and features a zigzag-shaped core with outwardly extending arms. Through N-glycosylation mapping and biophysical assays, it has been shown that uromodulin acts as a multivalent ligand for bacterial type 1 pilus adhesins, presenting specific epitopes on its arms. In vitro and patient urine sample studies revealed that uromodulin filaments bind to uropathogens, promoting bacterial aggregation, preventing adhesion, and facilitating elimination through micturition. Specifically, the high-mannose glycan at position N275 on uromodulin serves as the exclusive binding site for the bacterial type 1 pilus adhesin FimH of type 1-fimbriated *E. coli*. Moreover, when surrounded by polymers, this glycan induces pathogen aggregation and enhances its removal through urine excretion [77]. Uromodulin functions as a broad host defense factor, as it is effective against both *E. coli* and in infection models involving Proteus mirabilis [73], Klebsiella pneumoniae, Staphylococcus saprophyticus [72], and the fungal pathogen Candida albicans [78]. Interestingly, in the case of Candida albicans, uromodulin glycosylation did not affect binding. Additionally, uromodulin may have an immunomodulatory role by directly activating TLR4 and promoting the maturation of specific cell types, including myeloid dendritic cells [79]. 

Autoantibodies against uromodulin are believed to play a role in renal tubular disorders and urinary tract infections [14] and have also been detected in kidney transplant donors and recipients [80]. The frequency of the major allele of the UMOD promoter variant rs4293393, which is linked to increased uromodulin expression levels, exhibits a consistent correlation with pathogen diversity and the prevalence of antibiotic-resistant urinary tract infections in the population. Conversely, it demonstrated a negative correlation with urinary tract infection markers in the population [76].

Uromodulin plays a stabilizing immunomodulatory and anti-inflammatory role in the outer medulla of the kidney by crosstalk with adjacent proximal S3 tubules [81]. It suppresses the synthesis of cytokines and chemokines, including CXCL2 and IL-23 [15,82]. Beyond its role in modulating complex physiological processes and responding to stress signals, the concentration gradients of uromodulin also play a vital role in facilitating chemotaxis. It is probable that a uromodulin gradient also plays a significant role in maintaining the homeostasis of mononuclear phagocytic cells in the inner stripe of the outer medulla. This hypothesis is supported by findings in uromodulin knockout mice, where a decreased mononuclear phagocytic cell population was observed in this specific region. Interstitial uromodulin plays a crucial role in enhancing the abundance, adaptability, and phagocytic capabilities of mononuclear phagocytes [46]. Apart from its known impact on the epithelium, bone marrow granulopoiesis via IL23/17 signaling [83], and vascular calcification [84], uromodulin inhibits the production of reactive oxygen species (ROS) both within the kidney and throughout the body through inhibition of the transient receptor potential melastatin 2 (TRPM2) channel and the RAC1/JNK/c-JUN pathway [85]. Additionally, uromodulin may play a role in kidney injury and immune modulation by enhancing immunoglobulin and cytokine binding and activating monocytes and dendritic cells via Toll-like receptor 4 (TLR4) [60,61,79,86]. The presence of uromodulin within these lipid rafts suggests its potential intracellular functions in TAL cells [87]. However, the precise role of GPI anchoring [88], in addition to N-glycosylation [89], in facilitating the polarized trafficking of uromodulin to the apical membrane is not yet fully understood [43].

In the renal interstitium, uromodulin exhibits the ability to interact with multiple cell types present in this microenvironment. Uromodulin effectively suppresses pro-inflammatory signaling originating from the adjacent proximal tubules by targeting the basolateral domain of epithelial cells [15]. This includes the inhibition of neutrophil-chemokine release [90] and the production of interleukin (IL)-23, which stimulates the systemic release of IL-17 and enhances granulopoiesis via granulocyte colony-stimulating factor [46]. Consequently, fluctuations in uromodulin levels within the interstitium can be considered stress or danger signals that prompt a response from the surrounding cells [82]. Interstitial uromodulin plays a positive role in regulating the abundance, adaptability, and phagocytic activity of mononuclear phagocytes [46].

The influence of uromodulin on non-kidney organ systems appears to be closely linked to its circulation levels. Recent studies have revealed that serum uromodulin concentrations show an inverse correlation with inflammatory markers, such as C-reactive protein (CRP) and IL-1β, irrespective of kidney function, even in patients with normal kidney function [4,91,92]. Elevated serum uromodulin concentrations have been linked to a favorable metabolic profile, reduced prevalence of cardiovascular comorbidities, and a lower risk of 10-year mortality, independent of other cardiovascular risk factors, including eGFR, providing strong evidence for the positive impact of uromodulin on the homeostasis of the organism [93].

### 3.5. Analytical Aspects

Various methods are available for quantifying uromodulin in urine and plasma, including radioimmunoassays [51,52,54], enzyme-linked immunosorbent assays (ELISAs) [94,95], and high-performance liquid chromatography (HPLC) coupled to mass spectrometry (MS) [96,97,98]. To evaluate uromodulin levels in the circulation, a sensitive ELISA has been established, given that these levels are approximately 1000-fold lower than the levels found in urine [4]. Unlike immune-based approaches, MS-based techniques offer advantages by minimizing errors related to variable protein glycosylation, aggregation, or storage-mediated degradation. However, their suitability for high-throughput analysis has not been firmly established [99]. 

The structural integrity of uromodulin filaments can be influenced by factors such as the ionic strength of the solution, freezing, and storage time [47,49,51]. Both vortexing and centrifugation of urine samples significantly affected the detectable levels of uromodulin. To slow down degradation, urine samples are commonly stored at −80 °C. In large-scale biomarker studies, adherence to standard operating procedures is crucial for the reliable quantification of urinary uromodulin [94]. 

### 3.6. General Population

Creatinine and eGFR are traditional parameters influenced by muscle mass or diet and reflect renal function based on filtration ability. Unlike glomerular filtration, which has limited efficacy in representing overall kidney function, uromodulin provides substantial information about the overall kidney status. As a marker of tubular secretion, uromodulin provides valuable insights into the remaining nephron mass and thus reflects intrinsic kidney function beyond the scope of glomerular filtration alone. This breakthrough in diagnostics potentially addresses the issue of the creatinine-blind range associated with CKD, where kidney impairments often go undetected [53]. A pioneering large-scale study conducted on two Swiss population-based cohorts (SKIPOGH and CoLaus) revealed several significant associations: urinary uromodulin levels positively correlated with an eGFR < 90 mL/min/1.73 m^2^, urine volume, urinary electrolytes, and kidney volume. Conversely, they were negatively associated with age and diabetes mellitus [100]. These findings were further corroborated in a Canadian cohort (CARTaGENE), in which a strong association with fractional excretion of urate (FeUA) was observed [101]. The 24-h urinary excretion of uromodulin demonstrated a correlation with all known predictors of nephron mass, including birth weight, in both the healthy population and a group of kidney donors [102]. These comprehensive data suggest that urinary uromodulin serves as a valuable surrogate marker for assessing nephron-functioning mass in the general population. A plausible hypothesis suggests that low urinary uromodulin could serve as a marker of compromised tubular health, particularly in at-risk individuals such as those who are aged or have early-stage CKD. Decreased uromodulin levels may indicate reduced production, indicating diminished renal functional reserve. This hypothesis is supported by the study conducted by Garimella et al. [103], who followed 192 participants from the Cardiovascular Health Study over nine years. The results indicated that each SD increase in urinary uromodulin was associated with 23% lower odds of eGFR decline and a 10% lower risk of mortality. Similarly, lower preoperative urinary uromodulin levels have been linked to a higher risk of postoperative AKI [104]. In at-risk individuals, lower uromodulin levels might reflect a reduced functional reserve. 

In addition, serum uromodulin showed correlations with nephron mass, being absent in anephric patients, decreasing upon uninephrectomy, and increasing in transplant recipients depending on initial graft function [105]. Serum uromodulin is more sensitive than conventional markers that reflect glomerular filtration, such as serum creatinine, urea, and cystatin C, in detecting early-stage CKD [53,106]. Moreover, it has emerged as an early biomarker of fibrotic and atrophic kidney damage [107]. These findings highlight the potential of serum uromodulin as a valuable tool for the early detection and monitoring of kidney-related pathologies. Higher serum uromodulin concentrations showed an inverse correlation with progression to advanced kidney disease in elderly participants in the population-based KORA study. However, it did not offer any supplementary predictive value for CKD development in the same cohort [108]. According to the Cardiovascular Health Study, serum uromodulin was more strongly associated with eGFR than urinary uromodulin. Furthermore, the correlates of serum uromodulin and urinary uromodulin exhibited significant differences, indicating that the regulation of apical and basolateral secretions may vary distinctly [109]. However, in a separate small study cohort of 112 healthy living kidney donors, serum uromodulin concentrations were not correlated with the measured glomerular filtration rate (mGFR) and eGFR, which contrasts with the findings in patients with CKD [110]. In a study involving 3057 participants from the Ludwigshafen Risk and Cardiovascular Health study and 529 participants from the VIVIT study, the addition of serum uromodulin along with creatinine or cystatin C led to a more accurate risk model for all-cause and cardiovascular mortality [111]. This approach has the potential to enhance risk stratification. Patients who exhibited a decrease in eGFR while maintaining high serum uromodulin levels were more likely to be male and suffer from heart failure. This suggests that serum uromodulin may be involved in the enhanced tubular activation of the NKCL cotransporter, potentially contributing to heart failure. On the other hand, patients who showed a decrease only in serum uromodulin while maintaining high eGFR levels had a higher prevalence of coronary artery disease (CAD), an elevated fatty liver index, and increased triglycerides. These findings suggest that metabolic disturbances are key drivers of increased mortality in this group. By considering both eGFR and uromodulin levels, researchers may be able to distinguish different forms of cardiorenal syndromes based on their primary pathophysiological mechanisms. Additionally, this joint consideration of serum uromodulin and eGFR profiles reveals distinct intrinsic metabolic and clinical patterns, which could be significant indicators of kidney health and disease susceptibility, providing new insights into the understanding and management of kidney-related risks.

Through GWAS, genetic variants in *UMOD* have been associated with an increased risk of CKD and hypertension in the general population [24,112]. The primary *UMOD* risk variant (allele T at rs4293393), which is linked to both CKD and hypertension, is the ancestral allele. Its prevalence varies from 70–80% in Africans and Europeans and reaches approximately 90% in East Asians. Importantly, this risk variant directly enhances uromodulin expression in a dose-dependent manner, leading to salt-sensitive hypertension and kidney damage in both mice and humans. These findings highlight the significant impact of this variant on uromodulin levels and its implications for the development of hypertension and kidney-related conditions [113]. It is essential to recognize the possibility that *UMOD* single nucleotide polymorphisms (SNPs) may have additional effects unrelated to uromodulin production, which might be involved in the association with kidney disease [19]. Using rs12917707 as an instrumental variable in one-sample Mendelian randomization, higher urinary uromodulin was strongly associated with eGFR decline in a European population-based cohort of 3851 individuals. Using two-sample Mendelian randomization across four GWAS consortia demonstrated that higher urinary uromodulin levels were significantly associated with lower eGFR, heightened odds for eGFR decline or CKD, and elevated systolic and diastolic blood pressure. Specifically, each standard deviation (SD) increase in urinary uromodulin led to a reduction in log-transformed eGFR by −0.15 SD (95% CI: −0.17 to −0.13) and an increase in log-odds of CKD by 0.13 SD (95% CI: 0.12–0.15). One SD increase in urinary uromodulin resulted in a rise of 0.06 SD (95% CI: 0.03–0.09) in systolic blood pressure and 0.08 SD (95% CI: 0.05–0.12) in diastolic blood pressure. The effect of urinary uromodulin on blood pressure was mediated by eGFR, implying that changes in urinary uromodulin levels influence blood pressure through its impact on kidney function. In contrast, the effect of urinary uromodulin on eGFR was not mediated by blood pressure, suggesting that the adverse impact of genetically driven uromodulin levels on kidney function in the general population is a direct and causal relationship, independent of its effect on blood pressure. These findings strongly support the notion that genetically determined uromodulin levels play a key role in kidney function outcomes in the general population, with implications for understanding and managing kidney-related health issues [114]. 

The prevalence of the ancestral *UMOD* allele has remained high owing to its protective role against urinary tract infections, which significantly affects young women and has important consequences for fitness and reproduction. Additionally, the salt-retaining effect associated with this variant may confer an advantage to patients with severe infections, particularly in children. These evolutionary advantages likely contribute to the persistence of this allele in human populations [115]. The strong linkage disequilibrium observed among the top *UMOD* GWAS variants situated in the promoter region of the gene provides a crucial hint about their biological significance [113]. Population studies have further validated this insight by confirming a robust association between *UMOD* promoter variants and urinary levels of uromodulin. Notably, each major risk allele leads to an incremental increase in the urinary excretion of uromodulin in a dose-dependent manner [55,116]. Remarkably, individuals who are homozygous carriers of the risk allele exhibit uromodulin levels that are two-fold higher than those of homozygous carriers of the minor protective allele [55]. In conjunction with eGFR and sodium excretion, the *UMOD* genotype at rs4293393 has emerged as an autonomous predictor of urinary uromodulin levels, as demonstrated in a Canadian population [101]. Subsequent investigations revealed that the risk variants had a potent impact on the activity of the *UMOD* promoter in vitro. This effect was manifested by a two-fold increase in *UMOD* transcript levels observed in human kidney biopsies and a higher level of uromodulin present in urine. These data underscore the significant role of *UMOD* genetic variants in influencing uromodulin expression and secretion, shedding light on their potential implications for kidney function and related processes. Elevated uromodulin expression alone does not directly lead to kidney failure. Instead, it follows a second-hit model where prolonged high uromodulin production predisposes the kidneys to damage induced by additional comorbidities. In this context, other factors and underlying health conditions play a crucial role in contributing to kidney impairment over time, in conjunction with increased levels of uromodulin [113]. This model supports the robust interaction between age and the association of *UMOD* variants with kidney function [25,112]. Additionally, other factors that might contribute to potential harm include the metabolic demands of uromodulin production in TAL cells, the chronic activation of salt reabsorption systems, and the effects of increased uromodulin levels in the lumen of the distal nephron or circulating throughout the body. All these elements could collectively play a role in the development of kidney-related issues in individuals with elevated uromodulin expression [59]. In addition to contributing to kidney damage and aging-related lesions, the link between *UMOD* variants and hypertension has been demonstrated in a mouse model with increased uromodulin expression based on a gain-of-function mechanism [113]. This connection completes the picture, highlighting the association between the renal tubular transport of NaCl and blood pressure regulation. Taken together, these results provide valuable insights into the complex interplay between uromodulin, kidney function, and blood pressure regulation, shedding light on the potential mechanisms underlying hypertension [117].

Numerous studies have identified a significant association between the SNPs located within the *UMOD*-flanking gene *PDILT* and eGFR [118,119,120,121,122]. Specifically, rs77924615 was found to be linked to urinary uromodulin levels, but interestingly, it was not associated with *PDILT* expression. This observation suggests that *PDILT* likely serves as a causal regulatory variant, with *UMOD* as its effector gene. This hypothesis is supported by recent evidence indicating that *PDILT* acts as an expression-quantitative trait locus for *UMOD* [118].

## 4. Uromodulin and Acute Kidney Disease

**Figure 3:**
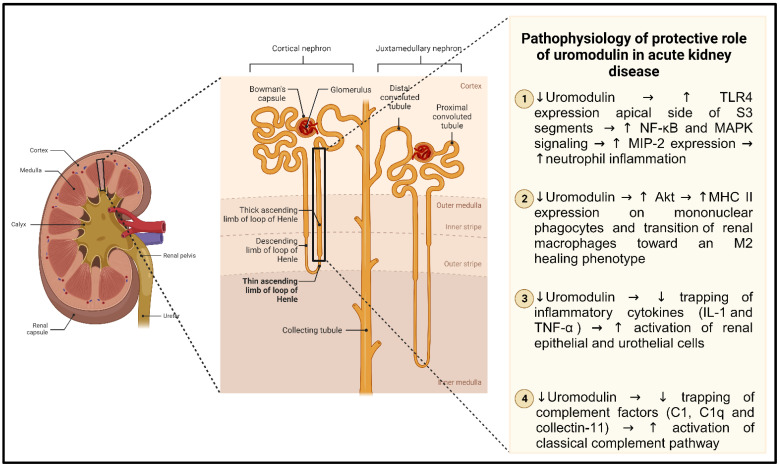
Overview of the role of uromodulin in acute kidney disease. Abbreviations: TLR4: Toll-like receptor 4; NF-κB: nuclear factor kappa-light-chain-enhancer of activated B-cells; MAPK: mitogen-activated protein kinase; MIP-2: macrophage inflammatory protein-2; Akt: protein kinase B; MHC II: major histocompatibility complex II; IL-1: interleukin-1; TNF-α: tumor necrosis factor-alpha; C1: complement factor 1; C1q: complement factor 1q.

Studies conducted in vivo on ischemia-reperfusion injury (IRI) models of AKI revealed heightened inflammation and necrosis in the kidneys of *UMOD* knockout mice, indicating a potential protective role of uromodulin (Table 1) [81]. Notably, the tubular cells that appeared to benefit from this protection predominantly belonged to the S3 proximal tubule segments, prompting the hypothesis of crosstalk between these cells and neighboring nephron segments [90]. During the recovery phase from IRI, uromodulin was found to relocate to the basolateral domains of the TAL and S3 epithelia, as well as in the interstitium. Ultimately, this process leads to the peak concentration of uromodulin in circulation [15]. However, the precise role of basolaterally released uromodulin in modulating inflammation remains unclear, and it is uncertain whether intermediates are involved in this process. Moreover, *UMOD* knockout mice exhibited a more severe phenotype after AKI, which has been linked to the impaired transition of renal macrophages toward an M2 healing phenotype. This suggests that uromodulin-mediated immunomodulation is related to the regulation of the number and activity of kidney mononuclear phagocytes. Interestingly, this phenotype could be improved by injecting truncated monomeric uromodulin, raising the possibility of using uromodulin modulation to counteract AKI [46]. The protective effect of uromodulin may also be linked to its ability to bind lymphokines, as demonstrated in both in vitro and in vivo studies of IL-1 and TNF-α [28,123]. Additionally, uromodulin can inhibit the activation of the classical complement pathway by binding to complement 1q [124] and collectin-11 [125]. Intriguingly, binding to collectin-11 is influenced by the uromodulin content of mannose and fucose, which is increased in AKI patients (Figure 3) [125].

Research conducted in two distinct populations, very-low-birth-weight infants [126] and elderly individuals with CKD [127], has revealed a significant link between lower initial levels of urinary uromodulin and an elevated risk of developing AKI. This correlation has also been validated in pediatric [128] and adult patients [104] undergoing cardiac surgery. AKI is a significant complication of on-pump cardiac surgery [129,130], and is primarily attributed to acute tubular necrosis [131]. Presently, preoperative evaluation of kidney function is confined to measures related to the glomerular axis, such as eGFR and the urinary albumin-to-creatinine ratio (ACR). Although various tubular injury biomarkers have been assessed for early post-surgery AKI diagnosis [132], routine preoperative assessment of tubular function remains uncommon. Two limited-scale studies involving patients undergoing cardiac surgery and critically ill patients, each with a sample size of less than 30 individuals, indicated that urinary uromodulin concentrations tended to decrease after AKI [133,134]. However, these studies did not investigate whether lower urinary uromodulin levels are associated with an increased risk of AKI. In another study involving 36 liver transplant recipients, individuals who developed AKI displayed lower pretransplant urinary uromodulin concentrations than those who did not experience AKI [135]. Furthermore, the urinary uromodulin levels of patients who did not develop AKI were similar to those of patients not undergoing liver transplantation. Additionally, in newborns admitted to the intensive care unit, low uromodulin levels were observed to serve as a predictive indicator of AKI [136]. These findings highlight the potential significance of uromodulin as a biomarker of AKI risk in different clinical settings. A retrospective analysis was conducted on a prospective cohort study comprising 218 adults who underwent on-pump cardiac surgery and showed a link between a decreased uromodulin-to-creatinine ratio and higher odds of AKI, although this association was not statistically significant after conducting fully adjusted analyses [104]. During a prospective cohort study involving 656 individuals hospitalized with AKI, urinary uromodulin levels were measured at various time points, from the time of diagnosis to 12 months after AKI. The findings revealed that an increase in urine uromodulin levels was associated with a 40% reduction in the risk of developing CKD [137]. Moreover, similar results were observed when examining serum uromodulin in a study involving patients with liver cirrhosis, where the admission level of serum uromodulin demonstrated an inverse relationship with the incidence of AKI during hospitalization [138]. Elevated serum levels of uromodulin have been detected in individuals diagnosed with acute tubular injury and/or acute interstitial nephritis [139]. However, measuring serum uromodulin did not provide a reliable means to predict the development of AKI during acute pancreatitis [140].

**Table 1:** Overview of studies investigating the role of uromodulin in acute kidney disease. Abbreviations: THP: Tamm-Horsfall protein; TLR4: Toll-like receptor 4; AKI: acute kidney injury; TAL: thick ascending limb; MIP-2: macrophage inflammatory protein-2; IFN-γ: interferon-gamma, IL1α: interleukin 1 alpha, TNF-α: tumor necrosis factor-alpha; IL6: interleukin 6, CXCL1: chemokine (C-X-C motif) ligand 1; IL13: interleukin 13; C3b: complement factor 3b; ICU: intensive care unit; VLBW: very low birth weight; CKD: chronic kidney disease; eGFR: estimated glomerular filtration rate; CPB: cardiopulmonary bypass; CCr: creatinine clearance rate.

Study Design	Study Population	Major Findings	Ref.
**Animal studies**			
	THP knockout mice vs. wild-type mice	THP^−/−^ mice had worse damage and impaired renal macrophage M2 transformation compared to wild-type mice.	[46]
	THP knockout mice vs. wild-type mice	THP^−/−^ mice showed more kidney inflammation and tubular necrosis. THP may stabilize the outer medulla by reducing inflammation, possibly affecting TLR4.	[81]
	THP knockout mice vs. wild-type mice	In AKI, S3 proximal segments suffer the most damage. TAL can affect S3 segment vulnerability by regulating THP-dependent MIP-2 expression.	[90]
	THP knockout mice vs. wild-type mice	The absence of THP significantly raised levels of circulating IFN-γ, IL1α, TNF-α, IL6, CXCL1, and IL13.	[123]
**Human studies**			
Cohort study	218 adults undergoing on-pump cardiac surgery	A preoperative decreased urinary uromodulin-to-creatinine ratio is linked to increased AKI odds and elevated peak serum creatinine after cardiac surgery.	[104]
Experimental study	10 normal human urine samples	THP seems to play a direct role in deactivating complement by serving as a cofactor for the breakdown of C3b.	[124]
Cohort study	20 ICU patients with AKI after surgery	Elevation of fucose levels on THP plays a significant role through the complement lectin pathway in AKI.	[125]
Cohort study	113 VLBW infants (weight ≤ 1200 g or < 31 weeks’ gestation)	Infants with AKI had lower minimum levels of urinary uromodulin on the first 4 postnatal days than those without AKI.	[126]
Cohort study	2351 CKD participants with 184 patients with an AKI event	Lower urinary uromodulin levels predicted future AKI, independently of eGFR and albuminuria.	[127]
Cohort study	101 children undergoing CPB	Children with the lowest pre-surgery urinary uromodulin levels faced a significantly higher risk of AKI following CPB.	[128]
Cross-sectional study	30 cardiac surgery patients divided into two groups of 15 each: group I without kidney dysfunction and CCr > 60 mL/min; and group II with CCr < 60 mL/min	Group II showed lower urinary excretion of uromodulin.	[133]
Cross-sectional study	21 ICU patients:. group 1 (*n* = 14) with no signs of kidney dysfunction vs. group 2 (*n* = 7) with the beginning of AKI	Patients who developed AKI exhibited reduced urinary excretions of uromodulin.	[134]
Cross-sectional study	14 liver transplant patients suffered kidney insufficiency, and 20 showed no AKI after liver transplantation	A higher pretransplant THP synthesis/urinary secretion may protect the kidneys during and after liver transplantation.	[135]
Case-control study	9 infants with AKI and 24 infants without AKI	Low urinary uromodulin levels may serve as a predictive indicator of AKI.	[136]
Cohort study	656 participants hospitalized with AKI	A rise in urinary uromodulin from baseline to 12 months was linked to a 40% lower risk of developing CKD.	[137]
Cohort study	98 patients with cirrhosis with subsequent hospital-acquired AKI	Reduced urinary uromodulin levels upon admission were linked to higher chances of later developing AKI in cirrhotic patients during hospitalization.	[138]
Case series	5 cases of patients with acute tubular injury and/or acute interstitial nephritis	Elevated serum uromodulin levels were found in patients with acute tubular injury and/or acute interstitial nephritis.	[139]
Cross-sectional study	66 adult patients with severe acute pancreatitis	While serum uromodulin correlated with kidney function in the early phase of severe acute pancreatitis, it did not reliably predict acute pancreatitis severity or AKI development.	[140]

## 5. Uromodulin and Chronic Kidney Disease

Plasma uromodulin is a reliable biomarker for assessing kidney function and offers a unique advantage in detecting the early stages of CKD. Unlike conventional renal retention markers, serum uromodulin demonstrates an inverse behavior, showing lower concentrations as kidney function declines [141,142]. Serum uromodulin levels exhibit superior discriminatory ability between non-CKD and CKD stage 1 compared to serum creatinine or eGFR alone [4]. In a landmark study by Risch et al. [142] involving 289 elderly individuals with varying stages of CKD, a positive correlation between serum uromodulin and kidney function was first demonstrated. Recent studies have further validated this positive association between serum uromodulin levels and kidney function in populations with kidney diseases of different etiologies [4,141]. There is a strong correlation between serum uromodulin and renal fibrosis [143]. In a prospective German chronic kidney disease study, elevated serum uromodulin levels were linked to a reduced risk of adverse kidney events over time [144]. In patients with albuminuria, serum uromodulin concentrations were notably reduced [91]. Lower serum uromodulin levels have been found to be predictive of an increased risk of developing ESKD [91,145,146]. Reduced urinary uromodulin levels have been shown to predict the development of CKD over a 9–10-year follow-up period [103], and the rapid decline of kidney function leads to the progression of ESKD in CKD patients within 1 year [147]. Patients diagnosed with CKD from different causes typically exhibit reduced urinary excretion levels of uromodulin, which shows a strong correlation with variations in eGFR [148]. *UMOD* promoter variants exhibit high associations with urinary levels of uromodulin, including both 24-h urinary excretion and the uromodulin-to-creatinine ratio, as observed in a meta-analysis involving 10,884 individuals of European descent [55].

A common variant in the *UMOD* promoter (rs12917707, minor T allele frequency 0.18) was the initial locus to be discovered in association with eGFR and CKD in individuals of European ancestry, and this association has already been established at a genome-wide significance level [24]. The presence of the minor allele of rs12917707 was independently associated with a 20% lower risk of CKD and a decreased risk of ESKD, irrespective of systolic blood pressure, hypertension medication, and diabetes mellitus. Numerous studies on populations with diverse ethnic backgrounds have replicated the association between *UMOD* promoter variants and renal traits [23,25,112,118,149,150,151,152,153,154]. A meta-GWAS conducted by the CKDGen Consortium, involving a total of 1,046,070 individuals with diverse ancestries, including European, East Asian, African American, South Asian, and Hispanic backgrounds, revealed that out of the 264 associated loci, the *UMOD* locus exerted the most significant impact on eGFR (OR: 0.81; 95% CI: 0.80–0.83; *p* = 3 × 10^−259^) [119]. *UMOD* SNP variants linked to kidney function have also been found to be associated with a decline in kidney function, including the incidence of CKD and ESKD [153,155,156]. Another association study focusing on CKD patients with different etiologies from the German CKD cohort demonstrated that the *UMOD* locus was also associated with advanced CKD [157]. Across all these studies, the protective effect of each copy of the minor allele of *UMOD* SNP rs12917707 ranged from a 24% to 59% reduction in risk. In the C-STRIDE Study, which involved 2731 Chinese patients with CKD stages 1–4, the rs11864909 variant in the *UMOD* gene was associated with both eGFR levels and serum uromodulin concentrations. Additionally, the variants rs4293393 and rs6497476 are associated with all-cause mortality in patients with CKD [158].

In two independent cohorts of the Framingham Heart Study and the Atherosclerosis Risk in Communities Study focusing on incident CKD, elevated urinary uromodulin concentrations preceded CKD onset. Notably, these urinary uromodulin concentrations were significantly associated with a CKD risk polymorphism located in the UMOD gene region, specifically rs4293393. The presence of each minor protective C allele at rs4293393 was associated with a CKD odds ratio of 0.76. Elevated urinary uromodulin levels were observed before the onset of CKD [116]. The observed higher levels of urinary uromodulin could be attributed to several factors, such as increased transcription, accelerated protein maturation, reduced efficiency of binding to its anchor, or enhanced cleavage into the urine. The association between elevated urinary uromodulin concentrations and incident CKD suggests that higher levels of uromodulin in urine might have detrimental effects, or it is possible that wild-type uromodulin serves a protective role within the cell before its secretion. Another plausible explanation is that the genetic variant may lead to modified glycosylation of uromodulin, potentially impacting its previously known functions, including its involvement in innate immunity [159]. This finding suggests that the presence of a polymorphism in the UMOD gene might be a contributing factor to the link between altered uromodulin concentrations and the development of incident CKD [116]. Of the 250+ loci associated with CKD and eGFR that have been identified, the genetic effect of UMOD appears to depend on age. Specifically, this effect is notably more pronounced in older individuals, particularly those aged > 65 years, and in the presence of age-associated comorbidities [25,149]. In contrast to what has been observed in AKI models, a lack of uromodulin appears to have a protective effect on CKD. Alleles linked to increased UMOD expression are associated with an elevated risk of CKD. Interestingly, these alleles provide protection against renal stone formation [160].

**Figure 4:**
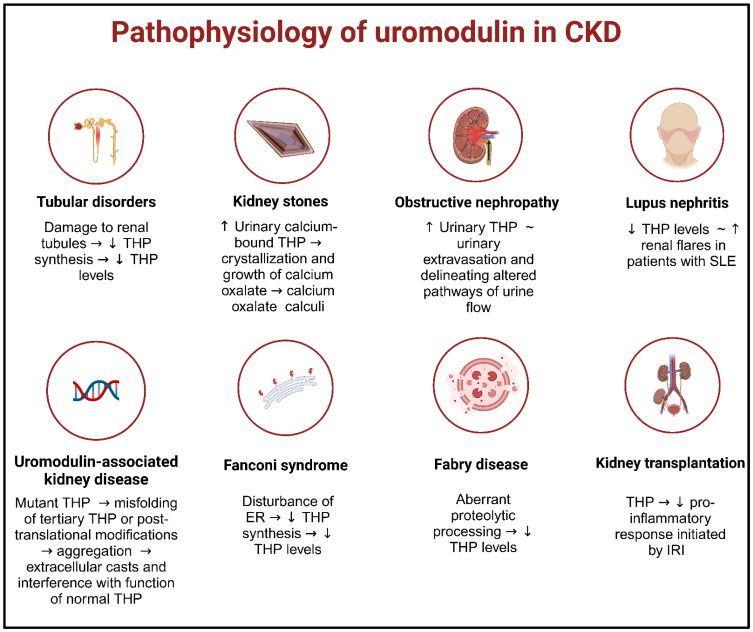
Overview of the role of uromodulin in chronic kidney disease. Abbreviations: CKD: chronic kidney disease; THP: Tamm-Horsfall protein; SLE: systemic lupus erythematosus; VUR: vesicoureteral reflux; ER: endoplasmic reticulum; IRI: ischemia-reperfusion injury.

In addition to its association with eGFR and CKD, uromodulin has been linked to other health conditions. For instance, in an extensive patient-control study conducted by the Global BPGen Consortium, it was found to be associated with the risk of hypertension and incident cardiovascular disease events [22]. Uromodulin has shown significant associations with uric acid levels in both an Icelandic cohort [25] and a Chinese community-based cohort [161]. Finally, in a combined sample of Icelandic and Dutch subjects, it has been linked to a reduced risk of kidney stones (OR: 0.88; 95% CI: 0.83–0.94) [25]. The prospective utility of uromodulin as a biomarker has been explored in various chronic diseases of distinct origins, which will be examined in the subsequent sections (Figure 4).

### 5.1. IgA Nephropathy

IgA nephropathy (IgAN) constitutes approximately 40% of primary glomerulonephritis cases and is characterized by hematuria, proteinuria, and a gradual decline in kidney function. It is distinguished by a diverse array of phenotypes and pathological alterations, with particular variations in tubular atrophy and interstitial fibrosis. When left untreated, it carries a significant risk, as 40% of patients may progress to ESKD within 10–20 years. Poor prognostic indicators for IgAN include hypertension, elevated serum creatinine levels, and increased urinary protein excretion [162]. 

In a Chinese pilot study of 344 IgAN patients, lower baseline urinary uromodulin levels (*p* = 0.03) and higher time-averaged proteinuria (*p* = 0.04) were identified as risk factors for rapid eGFR decline. Furthermore, urinary uromodulin correlated with tubulointerstitial lesions (*p* = 0.016). Patients with more pronounced tubular atrophy and interstitial fibrosis had lower urinary uromodulin concentrations (*p* = 0.02). Uromodulin is exclusively expressed by tubular cells at the corticomedullary junction, which is particularly susceptible to injury. Consequently, uromodulin synthesis and excretion may be affected by tubular atrophy and interstitial fibrosis in this region. Urinary uromodulin is an independent clinical factor associated with the progression of IgAN, and it potentially plays a dual role in CKD: damage to renal tubules may lead to reduced uromodulin synthesis, while uromodulin itself may also be involved in the pathogenesis of kidney disease progression. Decreased uromodulin excretion with tubulointerstitial lesions leads to weakened protection [163]. 

A retrospective single-center study of 180 patients with biopsy-proven IgAN found significant correlations between serum uromodulin levels and various kidney parameters. Serum uromodulin was positively associated with eGFR (*p* < 0.001, r = 0.5) and negatively correlated with serum creatinine (*p* < 0.0001, r = −0.51) and urinary protein (*p* = 0.005, r = −0.33). Patients in the low serum uromodulin group (<145 ng/mL) exhibited significantly higher serum creatinine levels (*p* < 0.0001) and more severe histopathological changes. Several prognostic factors for a 30% decline in eGFR were identified through univariate analyses, including male sex, elevated urinary protein levels, decreased serum albumin levels, low eGFR, and diminished serum uromodulin. In contrast to urinary uromodulin, low serum uromodulin emerged as a risk factor for severe histopathological changes. Furthermore, in contrast to other well-known prognostic factors for IgAN (eGFR, urinary protein, and hypertension), among patients in a relatively early stage of IgAN with eGFR > 60 mL/min/1.73 m^2^, multivariate analyses indicated that low serum uromodulin independently predicted a 30% decline in eGFR and the presence of severe histopathological changes. In the group with a higher degree of tubulointerstitial damage (T-scores of 1 and 2), both serum uromodulin and urinary uromodulin exhibited a significant reduction. The decrease in serum uromodulin levels might be attributed to disruptions in uromodulin production by distal renal tubular cells [162]. In conclusion, low serum uromodulin is associated with severe clinicopathological manifestations and could serve as a valuable serum marker for guiding therapeutic interventions in the early stages of IgAN. In the future, it is essential to gain a more comprehensive understanding of serum uromodulin dynamics in IgAN by conducting prospective studies involving a larger patient cohort.

ELISA-based detection of the urinary IgA-uromodulin complex proved to be a valuable non-invasive approach for diagnosing IgAN, with a sensitivity of 81.7%, a specificity of 73.4%, and an overall diagnosis efficiency of 78.2% [164]. Finally, ROC analysis of a fragment of uromodulin (*m*/*z* 1913.14) demonstrated excellent discriminatory capability, with an area under the curve (AUC) of 0.998 for distinguishing between IgAN and healthy controls. When differentiating IgAN from other glomerulopathies, the AUC remained strong at 0.815, indicating its potential as a reliable diagnostic marker [165]. Uromodulin has been included in several panels of biomarkers in IgAN patients. A urinary peptide classifier composed of fragments of various proteins (such as apolipoprotein C-III, alpha-1 antitrypsin, different collagens, fibrinogen alpha and beta, titin, hemoglobin subunits, sodium/potassium-transporting ATPase subunit gamma, uromodulin, mucin-2, fractalkine, polymeric Ig receptor, and insulin) demonstrated a significantly better predictive ability for the progressive loss of kidney function compared to using clinical parameters alone. The AUC for the urinary peptide classifier was 0.89 (95% CI: 0.83–0.95), while clinical parameters (including age, gender, proteinuria, eGFR, and mean arterial pressure) alone had an AUC of 0.72 (95% CI: 0.64–0.81). This highlights the potential of the urinary peptide classifier as a more accurate and sensitive tool for predicting the progression of kidney function decline in patients with IgAN [166]. Using discriminant analysis, another study found that a combination of seven markers, including three metabolites (dodecanal, 8-hydroxyguanosine, and leukotriene C4), three proteins (α_1_-antitrypsin, IgA-uromodulin complex, and galactose-deficient IgA1), and heparan sulfate, effectively differentiated patients with IgAN from those with other kidney diseases and healthy individuals [167]. 

### 5.2. Diabetic Nephropathy

Children/adolescents with type 1 diabetes exhibit serum uromodulin concentrations comparable to those of healthy controls [168]. Conversely, Wiromrat et al. reported reduced serum uromodulin concentrations in adolescents with type 1 diabetes compared with their non-diabetic counterparts. The reason for the decrease in serum uromodulin in patients with type 1 diabetes remains unclear. Lower serum uromodulin concentrations in individuals with type 1 diabetes are likely attributable to a change in uromodulin excretion rather than a reduction in renal tubular mass [169]. In addition to potentially indicating abnormalities in tubular mass, the reduced circulating uromodulin levels in adolescents with type 1 diabetes might also suggest the involvement of tubulointerstitial dysfunction in the development of kidney dysfunction. Low serum uromodulin concentrations have been linked to an elevated urinary ACR regardless of eGFR, suggesting an independent association between serum uromodulin levels and kidney function in this context [169,170].

Various studies have indicated that urinary uromodulin is initially normal following a diabetes diagnosis and then progressively increases with diabetes duration [95]. The uromodulin excretion rate remains unaffected by posture and increases during episodes of acute euglycemia and water intake [171]. However, in individuals with longstanding diabetes (>15 years) or advanced diabetic kidney disease (DKD), urinary uromodulin levels tended to decline [172,173]. Various studies have reported increased uromodulin excretion in the urine of adults with type 1 diabetes, particularly in the early stages of diabetes (duration < 15 years) [16,172,173,174]. Albuminuria correlated with reduced urinary excretion of uromodulin [175,176], and a decrease in urinary uromodulin levels was linked to an eight-fold increased risk of cardiovascular death and uremia [177]. 

Serum uromodulin levels seem to be associated with an increased risk of kidney tissue remodeling and potentially subsequent cardiovascular changes [168]. In type 1 diabetes, there is a pressing need for novel biomarkers that can more accurately predict coronary artery calcification (CAC), an indicator of subclinical atherosclerosis and DKD. Over the course of 12 years, the connections between serum uromodulin, CAC progression, and the development of DKD were examined in the coronary artery calcification in Type 1 Diabetes (CACTI) study (*n* = 527). A higher baseline serum uromodulin level was associated with reduced odds of CAC progression (OR: 0.68; 95% CI: 0.48–0.97), incident elevated albumin excretion (OR: 0.37; 95% CI: 0.16–0.86), rapid decline in GFR (OR: 0.56; 95% CI: 0.35–0.91), and impaired GFR (OR: 0.44; 95% CI: 0.24–0.83) for every 1 SD increase in serum uromodulin (68.44 ng/mL), after adjusting for baseline age, sex, systolic blood pressure, LDL cholesterol, and albuminuria/GFR. Moreover, the inclusion of serum uromodulin in models alongside traditional risk factors significantly enhances the predictive capability for CAC progression and the occurrence of DKD [178]. The association between low serum uromodulin concentration and CAC progression may be influenced or mediated by DKD [179,180]. However, Delgado et al. revealed a significant correlation between serum uromodulin concentration and the risk of cardiovascular mortality, regardless of eGFR, in patients undergoing coronary angiography. Serum uromodulin may actively contribute to the pathogenesis of atherosclerosis by directly exerting a significant role in suppressing the inflammatory and fibrotic pathways [93]. Atherosclerotic plaques in type 1 diabetes are believed to exhibit a higher degree of fibrosis and inflammation when compared to type 2 diabetes and the general population [181,182,183,184,185]. Consequently, factors such as serum uromodulin concentration, which influence vascular inflammation, may have significant clinical relevance in the context of type 1 diabetes. Based on these findings, it appears that serum uromodulin exhibits potential as a valuable tool for risk stratification and the prediction of cardiovascular disease and DKD development in adults with type 1 diabetes. Patients with low serum uromodulin concentrations may benefit from intensified control of vascular risk factors such as glycemia, dyslipidemia, and hypertension. Moreover, serum uromodulin concentration can also serve as an enrollment criterion for recruiting participants who are at a high risk of DKD and CAD progression, potentially enriching clinical trials with more events. Further research is imperative to fully comprehend the role of serum uromodulin concentration in DKD and CAD. Investigating the correlation between changes in serum uromodulin concentration and responses to existing and novel therapies will enhance our understanding of its pathogenesis [178]. 

Serum uromodulin also shows promise as a specific biomarker for early-stage DKD, developing in approximately 50% of genetically predisposed patients with type 2 diabetes [186]. Urinary extracellular vesicle uromodulin mRNA levels exhibited a gradual increase from type 2 diabetes mellitus to DKD groups, showing a correlation with commonly used diagnostic criteria such as eGFR and ACR. An extracellular vesicle mRNA signature has the potential to accurately identify DKD with a sensitivity exceeding 90% and a specificity of approximately 70% [187]. Uromodulin found in urinary microvesicles may be a specific marker for DKD and has the potential to be used for predicting the onset and/or monitoring the progression of DKD [188]. In patients with type 2 diabetes, the urinary glycated uromodulin concentration was markedly higher in patients with DKD than in non-diabetic CKD patients. The urinary glycated uromodulin concentration served as a predictor of DKD status, especially in patients with CKD stages 1–3a who were <65 years old and exhibited urine glycated uromodulin concentrations ≥ 9000 arbitrary units [189]. After undergoing Roux-en-Y gastric bypass, patients at risk for DKD may experience significant improvements in serum uromodulin levels, which appear to have a profound effect on restoring the structural integrity of nephrons [186]. 

Specific *UMOD* gene variants have been linked to distal tubular dysfunction, kidney function, and macroalbuminuria in adults with type 1 and type 2 diabetes [190]. A comprehensive population-based study comprising 133,413 individuals demonstrated that the association between *UMOD* promoter variants and eGFR based on serum creatinine level was most prominent among individuals with diabetes [106]. In a case-control study of 4888 unrelated type 2 diabetic subjects (including 880 with nephropathy and 4008 without nephropathy) from the Sweden-based Scania Diabetes Registry, the minor G allele at the *UMOD* variant rs13333226 was independently associated with a reduced risk of DKD. Additionally, this allele is associated with higher eGFR and lower systolic blood pressure [191]. A larger study encompassing type 1 and type 2 diabetes cohorts, involving over 40,000 subjects of European and Asian ancestry, further confirmed the association between *UMOD* variants and eGFR in individuals with diabetes [120]. However, it should be noted that some studies on Italians [192] and African Americans [193] did not replicate these findings, implying possible heterogeneity between different populations. The frequency of the *UMOD* rs4293393 variant with the C allele was notably higher in individuals with type 2 diabetes and DKD. This suggests that the *UMOD* rs4293393 T > C variation could potentially influence susceptibility to DKD in people of North Indian descent with type 2 diabetes [194].

### 5.3. Lupus Nephritis

Lupus nephritis (LN) is a significant systemic complication observed in individuals with systemic lupus erythematosus (SLE), with a reported prevalence of up to 70% and an incidence rate of 38% [195]. The projected 10-year occurrence of ESKD in individuals with LN is approximately 10% to 20% [195,196], accompanied by a standardized mortality ratio of 5.6 (95% CI: 3.7–7.5). The 10- and 20-year survival rates are notably reduced in LN [197]. Additionally, in a significant number of LN patients, the therapeutic response tends to be incomplete, with only 25–30% achieving prolonged remission [198]. Moreover, within two years after remission, approximately 24–45% of patients may experience new renal flares [199]. None of the current diagnostic markers (anti-dsDNA, complement factors C3 and C4, and more recently, anti-nucleosome antibodies) demonstrate high sensitivity in accurately indicating the presence of renal flares in all SLE patients, and ongoing efforts are focused on identifying new biomarkers for this purpose [200]. 

In the MRL-lpr/lpr mouse model, which mimics lupus tubulointerstitial nephritis and lupus glomerulonephritis seen in humans with SLE, two immune process-related molecules (uromodulin and β2-microglobulin) were identified in both urine and kidney tissues. When considering these biomarkers in combination and normalizing their expression by proteinuria level, they exhibited stronger predictive capabilities compared to relying on proteinuria determination alone. This highlights the potential of using uromodulin and β2-microglobulin together as a more effective and sensitive indicator for evaluating disease progression and severity in SLE-related kidney disorders [201]. 

In a cross-sectional study of 114 SLE patients, low serum uromodulin levels and a low serum uromodulin/eGFR index were correlated with higher scores of renal systemic lupus erythematosus disease activity index (SLEDAI), systemic lupus collaborating clinics (SLICC), kidney function, and proteinuria. SLE patients experiencing a renal flare demonstrated lower serum uromodulin levels than SLE patients without renal flares (*p* = 0.004). Unadjusted analysis revealed that serum uromodulin levels < 83 ng/mL were associated with a three-fold increased risk of renal flare. However, in the subsequent multivariable analysis, where serum uromodulin was adjusted for eGFR, the risk of renal flare based on the rSLEDAI was found to be 2.94, independent of other variables. The risk further rose to 4.27 when a patient with a flare had a SLICC renal disease activity score ≥ 5 [200]. Scherberich et al. provided further support for these findings through their analysis of patients with CKD. They found that individuals with SLE and kidney involvement exhibited lower serum uromodulin levels than patients with SLE without kidney involvement and controls [4]. After adjusting for potential confounding factors, having a low serum uromodulin/eGFR index (<0.80 ng/mL) significantly increased the risk of a renal flare (OR: 2.91; 95% CI: 1.21–6.98; *p* = 0.02) [200]. In contrast to other studies [4,202], diminished serum uromodulin concentrations showed a correlation with proteinuria severity. The correlation between lower serum uromodulin levels and proteinuria intensity remained evident even after adjusting for the eGFR using the serum uromodulin/eGFR index [200]. In conclusion, low serum uromodulin levels adjusted by eGFR could potentially serve as a marker associated with renal flares in patients with SLE. Nevertheless, to establish its clinical significance more conclusively, longitudinal studies that include patients with new-onset SLE and treatment-naive individuals are necessary.

In case-control studies, the urinary uromodulin level displayed the lowest mean value in LN patients, followed by SLE patients without LN and the control group [203,204]. Additionally, a positive correlation was observed between urinary uromodulin and eGFR, indicating its potential as a marker of kidney function. Conversely, a negative correlation was found between urinary uromodulin and serum creatinine, 24-h proteinuria, and SLICC renal activity scores. This suggests that lower urinary uromodulin levels may indicate kidney involvement and tubulointerstitial nephritis in patients with active SLE. Moreover, in the absence of activity markers, decreased urinary uromodulin levels may serve as an indicator of CKD and nephron loss. The measurement of urinary uromodulin levels can offer valuable insights into the extent of kidney damage in both active SLE and CKD cases, providing clinicians with an additional tool for assessing kidney health and disease progression [203].

### 5.4. ANCA-Associated Glomerulonephritis

Detecting biomarkers that offer prognostic or pathological insights independent of conventional factors, such as creatinine or eGFR, is crucial in patients with anti-neutrophil cytoplasmic antibody (ANCA) glomerulonephritis. Kidney biopsy in these patients is often challenging due to the increased bleeding risk. Therefore, identifying reliable biomarkers is particularly valuable for effectively managing their condition. 

A retrospective single-center study of 61 Japanese ANCA-associated glomerulonephritis patients revealed a positive correlation between serum uromodulin and urinary uromodulin, and both markers were correlated with kidney function. Serum uromodulin levels are positively correlated with eGFR. Patients in the high serum uromodulin group exhibited lower serum creatinine levels, focal classification, and milder tubulointerstitial injury than those in the low serum uromodulin group. However, there was no notable correlation between serum uromodulin concentrations and proteinuria. When comparing the characteristics among histopathological classes, patients in the focal class demonstrated the best kidney function and the highest urinary uromodulin-to-creatinine ratio and serum uromodulin. The focal class also showed significantly better kidney survival than the severe histopathological classes, namely the crescentic, mixed, and sclerotic classes. In univariate logistic regression analyses, low urinary uromodulin/creatinine, high serum creatinine, and low serum uromodulin were identified as prognostic factors for severe histopathological classes. In multivariate analyses, low serum uromodulin independently predicted the occurrence of severe histopathological classes, regardless of serum creatinine concentrations. The mean levels of serum uromodulin exhibited significant differences between the focal and severe histopathological classes, with a sensitivity of 70.6% and a specificity of 90.0% (using a cut-off value of 143 ng/mL and an AUC of 0.80). Nevertheless, low urinary uromodulin has not been identified as an independent risk factor for severe histopathological classes [205].

The decrease in serum uromodulin levels in patients with kidney dysfunction can be attributed to the disrupted production of uromodulin by renal tubular cells. Tubulointerstitial injury, which is prevalent in most cases of ANCA-associated glomerulonephritis, can significantly influence uromodulin production. No correlation was found between serum uromodulin and the N-acetyl-β glucosaminidase (NAG)/creatinine activity index or urinary β_2_-microglobulin levels. This may be due to the fact that circulating uromodulin originates from epithelial cells lining the TAL of the loop of Henle rather than from proximal tubules [205]. Although uromodulin interacts with various molecules and cells, such as complement factors, cytokines [206], IgG [163,207], T cells [28], neutrophils [208], and monocytes [159], the specific immunomodulatory effects of uromodulin in ANCA-associated glomerulonephritis remain unclear [205]. 

Based on the above findings, uromodulin serves as a highly valuable clinical parameter for predicting the severity of histopathological findings and could be regarded as a risk factor for progressive kidney dysfunction in ANCA-associated glomerulonephritis. Further research is essential to unravel the precise mechanisms underlying this phenomenon, and exploring the potential utilization of serum uromodulin levels as a novel prognostic biomarker in ANCA-associated glomerulonephritis warrants further prospective analysis.

### 5.5. Kidney Stones

Renal calculi disease, also known as kidney stone disease, is the most prevalent urological disorder in both men and women, with a slightly higher incidence in men. The lifetime likelihood of an individual developing kidney stones is approximately 10%, whereas the risk of recurrence within a 10-year period is as high as 74% [209].

Several case-control studies have consistently demonstrated reduced urinary uromodulin levels in individuals with kidney stone formation [97,210,211]. After isolation of uromodulin from urine samples, kidney stone patients exhibited significant differences in their biochemical composition compared to the controls. Specifically, protein content was 32% lower in patients with kidney stones than in the control group. Additionally, sialic acid content was reduced by 29% in male kidney stone patients and 24% in female kidney stone patients when compared to controls. Moreover, the neutral and amino sugars were also notably lower in kidney stone patients, showing an 18% decrease in males and a 20% decrease in females compared to the control group. All observed differences between patients with kidney stones and controls were statistically significant (*p* < 0.001). These findings shed light on the potential role of altered biochemical profiles in kidney stone formation [212]. The desialylation of uromodulin seems to nullify its normal defensive function by triggering protein aggregation, which, in turn, leads to the aggregation of calcium oxalate monohydrate crystals. This creates a favorable environment for the formation of kidney stones [213]. 

However, other studies suggested that uromodulin, owing to its presence in kidney stones, is involved in the formation of renal calculi [214,215]. Individuals with a strong family history of severe recurrent calcium stone formation had an increased excretion of uromodulin compared with healthy controls [216]. The oxidized forms of uromodulin play a significant role in promoting the crystallization and growth of calcium oxalate crystals, which are crucial processes in the formation of kidney stones composed of calcium oxalate [217]. 

In vitro studies using models of calcium oxalate-induced toxicity in renal epithelial cells have yielded conflicting results regarding the role of uromodulin in crystal growth and aggregation [218]. Systematic analyses showed that uromodulin at concentrations ranging from 0.4 to 40 μg/mL exhibited a concentration-dependent reduction in the size of calcium oxalate monohydrate crystals during initial crystallization without affecting the crystal mass. As crystallization progressed, uromodulin showed concentration-dependent inhibition of calcium oxalate monohydrate crystal growth and aggregation. At the highest concentration (40 μg/mL), uromodulin prevented the adhesion of crystals to cells. However, it did not affect crystal invasion through the extracellular matrix. Sequence analysis of human uromodulin revealed the presence of two large calcium-binding domains (residues 65–107 and 108–149) and three small oxalate-binding domains (residues 199–207, 361–368, and 601–609). These findings shed light on the molecular mechanisms by which uromodulin can influence the formation and growth of calcium oxalate monohydrate crystals, providing potential insights into the prevention of kidney stone formation. Investigations into the calcium-affinity and/or oxalate-affinity of uromodulin revealed that uromodulin exhibited a strong affinity, specifically with calcium, but not with oxalate. Through functional validation, it was further confirmed that when uromodulin was saturated with calcium (but not with oxalate), it eliminated the inhibitory effects of uromodulin on calcium oxalate monohydrate crystal growth, aggregation, and crystal-cell adhesion. This highlights the importance of calcium binding by uromodulin in mediating its regulatory impact on the formation and interactions of calcium oxalate monohydrate crystals [219]. To gain more conclusive insights, in vivo experiments were conducted using *UMOD*-KO mice. These mice showed spontaneous intrarenal crystallization that was unaffected by sex or genetic background. The severity of this phenotype increased with age, and it displayed gene dosage dependence, with heterozygous mice exhibiting intermediate severity [220,221,222]. 

Citrate plays a vital role in determining the rates of calcium oxalate crystallization and crystal morphology, regardless of the presence of native uromodulin or stone-forming uromodulin [218,223]. Its influence appears to outweigh that of uromodulin, as even stone-forming uromodulin, known to promote crystallization, becomes a crystallization inhibitor in the presence of citrate. This emphasizes, at a morphological level, that what has been observed in functional and clinical studies is that urine requires equimolar concentrations of citrate and calcium to prevent the formation of large crystal aggregates in the presence of abnormal uromodulin [218].

A GWAS reported an association between the major allele of the *UMOD* SNP rs4293393 (linked with higher uromodulin expression) and a decreased risk of kidney stone formation (OR: 0.88; 95% CI: 0.81–0.96; *p* = 0.0053) [25], which was also supported by the findings of Patel et al. [224]. These findings suggest that lower levels of uromodulin in the urine promote the formation of kidney stones. Additionally, it is worth considering that the protective effect of uromodulin against kidney stones might be indirectly mediated by enhancing calcium reabsorption in the DCT through the activity of TRPV5/6 channels, thereby reducing luminal calcium concentration [63]. 

To recapitulate, the prevailing body of genetic and in vivo evidence strongly substantiates the notion that uromodulin primarily acts as a potent inhibitor in the process of kidney stone formation, despite a few studies that may suggest otherwise. However, the exact mechanism of action and the specific stage of crystal formation at which uromodulin exerts its effects require further elucidation [160].

### 5.6. Obstructive Nephropathy

Urinary tract obstruction is a prevalent urological issue that can arise from various diseases affecting individuals of all age groups. Although obstructive uropathy leading to obstructive nephropathy may not be the most common cause, it remains a significant contributing factor to the development of CKD [225]. Chronic obstruction is known to induce injury in the tubular and interstitial regions of the kidney through the activation of intrarenal angiotensin II. This activation subsequently leads to the release of cytokines and adhesion molecules, which in turn trigger macrophage infiltration, the generation of reactive oxygen species, and a reduction in renal blood flow [226,227]. In the context of obstructive nephropathy, an increase in intratubular hydrostatic pressure exerts its pathogenic effects through three mechanisms: (1) induction of tubular ischemia due to reduced blood flow; (2) mechanical stretching or compression of tubular cells caused by elevated pressure; and (3) alteration of urinary shear stress in the affected tubules [228]. Prolonged urinary tract obstruction can lead to renal parenchymal atrophy. Unfortunately, symptoms often become apparent only after the chronic obstruction has already caused a partial reduction in kidney function [229]. Biomarkers of kidney function can be beneficial for the early detection of kidney failure. Although the serum creatinine level is commonly used to assess overall kidney function, it is not sensitive enough to detect early changes in GFR or unilateral kidney damage. Therefore, additional sensitive biomarkers are necessary for the early detection of kidney dysfunction [230].

In a rat model of unilateral obstructive uropathy, urinary casts containing uromodulin were identified not only downstream from the location of the uromodulin synthesis within the cells of the ascending limbs of the loop of Henle, but also at other points along the urinary tract. While uromodulin is not directly implicated in the pathogenesis of tissue injury in obstructive uropathy, it serves as a valuable marker for detecting urinary extravasation and delineating the altered pathways of urine flow within the kidney during pathological conditions [231]. 

In a cross-sectional study of 57 CKD patients based on obstructive nephropathy, serum uromodulin was evaluated as a marker of early kidney dysfunction, measuring at 50.2 ± 26.3 ng/mL in comparison to the control group, which had a level of 78.3 ± 24.5 ng/mL (*p* < 0.001). Variations in serum uromodulin levels were observed among groups with distinct renal function statuses. A positive correlation was found between serum uromodulin and both measured GFR (r = 0.757, *p* < 0.001) and effective renal plasma flow (r = 0.572, *p* < 0.001), with lower serum uromodulin levels observed in patients with impaired kidney function. Conversely, an inverse relationship was identified between serum uromodulin and various filtration markers such as cystatin C, creatinine, urea, and uric acid. ROC analysis of serum uromodulin for distinguishing between obstructive nephropathy patients with a GFR < 60 mL/min/1.73 m^2^ and those >60 mL/min/1.73 m^2^ resulted in an AUC of 0.98 (*p* < 0.001, 95% CI: 0.922 vs. 0.998) with a cut-off value of 46 ng/mL, demonstrating a specificity of 96.8% and sensitivity of 92.2% [232].

### 5.7. Vesicoureteral Reflux 

Vesicoureteral reflux (VUR) is a common congenital abnormality affecting the urinary tract [233,234]. This condition leads to urinary tract infections [235], which can result in permanent damage called reflux nephropathy, characterized by renal scars. The consequences of reflux nephropathy include hypertension, pregnancy complications, and kidney failure [236,237]. The degree of kidney damage is correlated with both the severity of reflux and the frequency of urinary tract infections, although other factors may also contribute to this condition [234]. 

The kidneys of animals and patients with focal scarring secondary to VUR frequently exhibit interstitial uromodulin deposits [238]. The pathogenetic mechanism leading to chronic pyelonephritis may involve an autoimmune response directed against uromodulin. The production of antibodies can be triggered by various factors, such as bacteriuria, which collectively contribute to the development of the condition [238,239]. Histological evidence of interstitial mononuclear cell infiltration and fibrosis was found in 10 children with these deposits in kidney allografts. Of these patients, 80% had VUR in the graft and a history of urinary tract infections [240]. In a study of 26 consecutive patients with primary VUR, low urinary uromodulin excretion was associated with decreased kidney function [241]. 

A negative relationship has been observed between urinary levels of uromodulin and urinary tract infection markers [115]. In a prospective cohort study involving elderly individuals living in the community, those with higher urinary uromodulin concentrations in the top quartile had a reduced risk of urinary tract infection events compared to those in the lowest quartile. Remarkably, this protective effect of uromodulin remained significant even after accounting for classical urinary tract infection risk factors [69].

Children diagnosed with VUR who possess the *UMOD* genotype rs4293393 TC appeared to have a higher susceptibility to developing renal scars, irrespective of any prior history of febrile urinary tract infections [242]. Individuals who are homozygous carriers of the *UMOD* risk T allele (T, major) at rs4293393 exhibit two-fold higher levels of uromodulin in their urine than those who are homozygous carriers of the (C, minor) protective allele [243]. 

### 5.8. Uromodulin-Associated Kidney Diseases

Mutations in the *UMOD* gene underlie several kidney diseases, such as MCKD2, FJHN [20], and glomerulocystic kidney disease with hyperuricemia [244,245]. These conditions are collectively known as uromodulin-associated kidney diseases (UAKD). UAKD represents a group of autosomal dominant disorders characterized by various clinical manifestations, including alterations in the urinary concentrating ability, tubulointerstitial fibrosis, hyperuricemia, and gout. In some cases, renal cysts may be present at the corticomedullary junction [21,246,247]. Although UAKD exhibit heterogeneity in terms of age at onset, clinical features, and presence of cysts, they invariably lead to the development of CKD during the third to fifth decades of life [248]. While the presence of corticomedullary cysts is well documented in MCKD2, their occurrence in FJHN has not been extensively recorded [249]. The clinical manifestations of MCKD2 and FJHN can vary in terms of their presence and severity, making the diagnosis challenging, particularly in milder cases [249,250]. 

Of the total *UMOD* mutations identified, 60% affected one of the 48 conserved cysteine residues [251]. Mutations in the *UMOD* gene may lead to disruption in the tertiary structure of uromodulin, which is responsible for the clinical manifestations of interstitial renal disease, polyuria, and hyperuricemia observed in both MCKD2 and FJHN [20,252]. The expressed mutant proteins exhibited unique glycosylation patterns, disrupted intracellular trafficking, and reduced capability to be exposed on the plasma membrane, resulting in reduced urinary uromodulin excretion and a case-specific uromodulin immunohistochemical staining pattern in the kidney [244,251,253]. Individuals carrying two *UMOD* mutations are viable but tend to experience more severe disease on average compared to heterozygotes [254]. 

Abnormal uromodulin isoforms are found in the urine of UAKD patients with preserved kidney function. These mutant uromodulin variants have a higher tendency to aggregate compared to the normal (wild-type) protein due to misfolding or differences in post-translational modifications, such as glycosylation. The aggregation of these abnormal isoforms may lead to the formation of extracellular casts, causing tubule obstruction and interfering with the function of both wild-type uromodulin and possibly other membrane proteins. These findings indicate that the proteotoxic effect of mutant uromodulin isoforms may be driven by both intra- and extracellular gain-of-function mechanisms [248].

Patients with FJHN and kidney insufficiency display a significant decrease in urinary uromodulin levels, accompanied by either elevated or decreased plasma uromodulin levels. Distinct urinary and plasma uromodulin profiles have been observed in the early and late stages of the disease, suggesting an altered direction of uromodulin secretion during the course of FJHN. This alteration could be attributed to improper intracellular sorting of the mutated protein in the TAL of the kidney [255]. Uromodulin excretion is characterized by a progressive decline from low normal values during childhood to extremely low levels in early adulthood in individuals with FJHN [256]. In patients with FJHN, a hyperplastic endoplasmic reticulum has been observed in the TAL of the loop of Henle. Additionally, an overexpression of endoplasmatic reticulum-resident molecular chaperones such as BiP (immunoglobulin binding protein) or PDL has been reported [253,257,258], suggesting an exaggeration of the unfolded protein response [259,260]. The *UMOD* mutations do not hinder the GPI-mediated apical targeting of the protein. However, they affect the apical secretion of proteins [255]. The main driver of kidney damage in FJHN is believed to be the pathological accumulation of mutant uromodulin protein [261,262]. As a result, chemical chaperones such as colchicine or phenylbutyrate have been found to stabilize mutant proteins and alleviate cellular damage caused by *UMOD* mutations [261]. 

In 2002, Hart et al. reported that dominant mutations in *UMOD* play a significant role in the development of ADTKD, a rare condition often accompanied by normal urinalysis and tubulointerstitial damage, with slowly progressive CKD usually first noted in teenagers and progressing to ESKD between the third and seventh decades. Hyperuricemia is frequently observed from an early age, and gout, arising from reduced kidney excretion of uric acid, develops in approximately 8% of affected individuals during their teenage years. Over time, this condition progresses, and gout eventually affects approximately 55% of affected individuals [20,263]. The genetic basis of this disease is diverse, involving multiple genes that contribute to its genetic heterogeneity. Mutations in mucin 1 (MUC1), hepatocyte nuclear factor-1 beta (HNF-1β), renin (REN), and the alpha 1 subunit of translocon 61 (SEC61A1) have been identified as additional causative factors for the development of this condition [264]. *UMOD* mutations can invariably lead to CKD and kidney failure [260,261,262,263]. In contrast to canonical ADTKD mutations, individuals carrying the p.Thr62Pro mutation exhibited milder disease severity, showing slower CKD progression. Moreover, there was an intermediate reduction in urinary uromodulin levels, consistent with an intermediate trafficking defect observed in vitro, along with a relatively modest induction of endoplasmic reticulum stress [265]. The mechanism of action of *UMOD* mutations is mostly a gain-of-toxicity function. The majority of UMOD mutations reported to date are missense changes that frequently involve the replacement or deletion of conserved cysteine residues within the protein. This alteration affects the proper folding and trafficking of the uromodulin protein to the plasma membrane. In vitro studies have demonstrated this effect, while analysis of patient biopsies has revealed the characteristic accumulation of mutant uromodulin in the endoplasmic reticulum [251]. These observations confirm that ADTKD-UMOD is indeed a form of endoplasmic reticulum storage disease [257,266]. *UMOD* mutations are characterized by a distinctive trait: reduced fractional excretion of uric acid (FeUA), resulting in hyperuricemia that precedes CKD and gout. In a recent study, a clinical uromodulin-score score was created, which incorporates the specific clinical features of various ADTKD subtypes along with urinary uromodulin levels. This scoring system serves as a valuable tool for prioritizing the genetic testing of affected individuals [267]. In line with its retention in the endoplasmic reticulum, uromodulin levels in the urine and blood of individuals with ADTKD-*UMOD* exhibit a significant reduction [268,269]. When comparing heterozygote and homozygote mutation carriers, a gene dosage effect was observed, with the homozygote proband showing remarkably low urinary levels of uromodulin and the presence of abnormal uromodulin fragments in the urine [270]. 

*UMOD* mutations result in specific transport alterations characterized by an exaggerated response to furosemide and an inability to maximally concentrate urine during the early phase of the disease [271]. In a Tg^UmodC147W^ mouse model, it was demonstrated that young Tg^UmodC147W^ mice exhibited upregulation of inflammation and fibrosis, as well as downregulation of lipid metabolism, even before any functional or histological evidence of kidney damage was apparent. In ADTKD-*UMOD*, proinflammatory signals appear before the onset of fibrosis and are detectable in the first week after birth. Early induction of inflammation is likely crucial for the pathogenesis of ADTKD-*UMOD*, suggesting that related pathways could serve as potential novel targets for therapeutic intervention [272].

### 5.9. Autosomal Dominant Polycystic Kidney Disease

Autosomal dominant polycystic kidney disease (ADPKD) is the most prevalent hereditary kidney disorder that can advance to ESKD. It is characterized by enlarged kidney volume caused by cyst formation. In a study of 54 patients with ADPKD and 18 healthy volunteers, the serum uromodulin level was significantly lower in the healthy volunteers than in the ADPKD patient group (*p* = 0.021). There was a correlation between serum uromodulin levels and eGFR, suggesting a potential cause-and-effect relationship. A simple linear regression analysis was conducted to evaluate the relationship between serum uromodulin levels and eGFR, and the obtained R^2^ value was 0.075. This indicates that approximately 7.5% of the variance in serum uromodulin values corresponded to the change in eGFR value. Nevertheless, it is essential to consider that abnormalities in tubular cells could potentially lead to irregular uromodulin secretion [273]. The total kidney volume was negatively correlated with urinary uromodulin levels [274].

### 5.10. Kidney Transplantation

Delayed graft function (DGF) is typically characterized by the requirement for dialysis within a week following kidney transplantation. It affects approximately 25–50% of patients and is linked to an elevated risk of acute rejection episodes and diminished long-term graft survival [275,276,277,278]. Histologically, DGF primarily manifests as severe IRI with inflammatory tubular damage [279]. IRI initiates a prolonged inflammatory response, resulting in interstitial fibrosis and tubular atrophy, ultimately compromising the overall graft survival [280,281,282]. Enhancing our understanding of the pathophysiology of IRI and exploring ways to target it could lead to improvements in long-term kidney graft survival [283]. However, available measures to mitigate IRI and predictive markers for DGF before transplantation remain scarce and have limited diagnostic value [284].

In a single-center prospective observational cohort study with 239 kidney transplant recipients, multivariable logistic regression analysis showed that after adjusting for the recipient, donor, and transplant-associated risk factors, each 10 ng/mL increase in pretransplant serum uromodulin was linked to a 47% lower likelihood of DGF (OR: 0.53, 95% CI: 0.30–0.82) [285]. Elevated pretransplant serum uromodulin might signify the recipient’s increased anti-inflammatory capacity to counteract inflammation caused by IRI. Studies have demonstrated that interstitial or serum uromodulin can downregulate proinflammatory signaling in the kidney, highlighting its immunomodulatory and reno-protective potential [46]. It is intriguing to note that serum uromodulin levels show an initial increase in patients, regardless of whether they experience DGF or not, which could indicate the release of “donor” serum uromodulin from the transplanted kidney. However, in patients with DGF, there was a subsequent significant and sustained decrease in serum uromodulin. When dividing pretransplant serum uromodulin into quartiles, the lowest quartile had a 4.4-fold higher likelihood of DGF compared to the highest quartile (OR: 4.41, 95% CI: 1.54–13.93). Incorporating pretransplant serum uromodulin into a model with established risk factors for DGF during multivariate ROC curve analysis improved the AUC from 0.786 (95% CI: 0.723–0.848) to 0.813 (95% CI: 0.755–0.871, *p* = 0.05). However, serum uromodulin on postoperative day 1 did not show any association with DGF [285]. Reduced levels of serum uromodulin can be detected during the initial stages of tubulointerstitial injury in kidney transplants [286]. The absence of an association between serum uromodulin on postoperative day 1 and DGF may indicate dynamic and complex pathophysiological processes occurring during the early post-transplant period in the kidney. This critical time frame may influence the subsequent course of injury or recovery. The initial increase in serum uromodulin levels could potentially signify a generalized reactive renoprotection response, where uromodulin production is induced in response to renal IRI, highlighting its immunomodulatory capabilities within the interstitium [15,81]. After transplantation, patients who did not experience DGF maintained elevated serum uromodulin levels over the long term. In patients who developed DGF, a subsequent decline in serum uromodulin during the postoperative period was observed [285]. Serum uromodulin levels on postoperative day 1 might be affected by acute inflammation and hypoxic stress, while serum uromodulin over the long term is more likely to reflect tubular function or mass [92,287]. 

In summary, elevated pretransplant serum uromodulin levels were independently associated with reduced odds of DGF. This suggests that serum uromodulin could potentially serve as a noninvasive marker, allowing for patient stratification based on the risk of developing DGF shortly after kidney transplantation [285]. In cases where graft function failed due to acute tubular necrosis, urinary uromodulin levels exhibited a significant reduction compared to levels observed in urine produced during acute rejection episodes (*p* < 0.01) or during periods of stable graft function (*p* < 0.02). This observation suggests that urinary uromodulin levels may serve as an indicator of tubular damage rather than being influenced by other causes of graft functional failure [288].

Uninephrectomy in healthy individuals results in a significant increase of approximately 40% in the excretion of uromodulin from the remaining kidney in the donor. Interestingly, in the transplanted kidney, the uromodulin excretion rate remained unchanged. Furthermore, the uromodulin excretion rate was correlated with graft failure. The exact mechanism behind this association is currently unknown because uromodulin does not undergo glomerular filtration [289]. A study of 100 kidney transplant patients showed that the serum levels of uromodulin exhibited a gradual decrease, starting in individuals with relatively well-preserved graft function and reaching the lowest levels in kidney transplant recipients with predialysis CKD. Serum uromodulin exhibited significant correlations with all parameters of kidney graft function, with the most robust association observed for eGFR CKD-EPI creatinine-cystatin C. This was followed by serum levels of urea, eGFR CKD-EPI creatinine, serum levels of cystatin C, eGFR CKD-EPI cystatin C, serum levels of creatinine, and ^51^CrEDTA. In the initial phases of CKD after transplantation, a decrease in serum uromodulin was observed even before other markers, such as serum creatinine and precise GFR determination with ^51^CrEDTA, fell outside the reference range. This suggests that the reabsorption of uromodulin, which is exclusively produced by tubules, may be compromised during the early stages of tubulointerstitial injury. Consequently, reduced serum uromodulin could potentially serve as a more sensitive indicator of early kidney graft dysfunction, which is not detectable using serum glomerular filtration markers. Considering the simplicity and cost-effectiveness of measuring serum uromodulin concentrations, monitoring its dynamics may prove to be a valuable, non-invasive predictive biomarker for assessing kidney graft injury and its outcomes. In the context of kidney transplantation, serum uromodulin is of particular value for clinicians who appreciate the benefits of biomarkers that reflect subclinical tubular injury. Incorporating regular serum uromodulin checkups for kidney transplant recipients could be a valuable approach for the early detection of subclinical processes affecting the tubules and interstitium, including acute tubulointerstitial rejection, as well as for timely monitoring of interstitial fibrosis and tubular atrophy (IF/TA) [286].

Presently, there are no reliable methods available to monitor tubular function, as conventional biomarkers solely reflect glomerular filtration. From a pathophysiological perspective, the prospect of monitoring tubular function seems promising, especially in diseases characterized by predominant tubular injuries such as BK-nephropathy [290]. As an indicator of renal tubular function, uromodulin demonstrated predictive capabilities for graft loss similar to conventional markers of glomerular filtration, including creatinine, blood urea nitrogen (BUN), eGFR, and cystatin C. An increase in serum uromodulin by one SD was linked to an impressive 80% reduction in risk. Moreover, when comparing the average uromodulin concentrations between the lowest and highest quartiles, a hazard ratio of 0.02 indicated a remarkable 98% risk reduction for the group in the highest quartile. Using ROC analysis, a threshold value of 24.0 ng/mL for uromodulin was determined, resulting in a sensitivity of 90% and a specificity of 70% for accurately predicting graft loss. When combining uromodulin and eGFR in a risk stratification model for graft loss, the predictive accuracy was significantly improved compared to models containing only one of these parameters. Monitoring tubular function in transplant recipients provides valuable insights into kidney function, and the decline in kidney transplant function affects both glomerular and tubular functions simultaneously. Despite the novel insights it provides into the pathophysiological understanding of kidney transplant function, it is premature to recommend the implementation of uromodulin into the clinical routine. Further research and validation are necessary to establish their utility as reliable clinical biomarkers [92].

Late kidney transplant failure is primarily caused by the progression of fibrosis resulting from the chronic alloimmune response and side effects of immunosuppressants [291,292]. Monitoring the progression of graft fibrosis can be effectively achieved through mGFR. However, this process can be time-consuming and may not be suitable for routine clinical practice. Therefore, there is a demand for noninvasive biomarkers that accurately reflect tubular health, enabling early detection of the accelerated development of fibrosis with precision comparable to that of the mGFR [293]. A post hoc analysis of the Omega-3 fatty acids in Renal Transplantation (ORENTRA) trial showed an association between serum uromodulin and mGFR at the end of the study (1 year after transplantation), but not at baseline. Serum uromodulin was inversely associated with the graft interstitial fibrosis (IF%) score. One possible explanation for this observation is that serum uromodulin requires time to reach a steady state in circulation after being released from tubular epithelial cells [294]. Experiments focusing on urinary excretion have estimated that uromodulin has a half-life of approximately 16 h [54], but it could vary significantly, ranging from 3 h to 7 days [295]. However, few studies have investigated the turnover rate of circulating uromodulin. At 8 weeks post-transplant, kidney graft function may not have been fully stabilized for all patients, and as a result, not all patients would have reached a steady state in their serum uromodulin levels by that time. This variability in serum uromodulin concentration could influence the observed associations with the graft IF% score [294]. Serum uromodulin holds potential as a valuable marker in the follow-up of kidney transplant recipients for detecting the early stages of fibrosis development.

Deceased donors experience significant biological changes where the injury and recovery processes are simultaneously activated in response to ischemia [296]. Following brain death, neurogenic hypotension and systemic upregulation of proinflammatory markers occur. These factors contribute to direct kidney injury due to ischemia and reperfusion, leading to the activation of inflammatory pathways [297,298,299]. In addition to injury and inflammation, the kidney initiates both adaptive and maladaptive processes [300,301]. Kidney transplantation presents a unique scenario in which the immunomodulatory properties of uromodulin may lead to a maladaptive response. Interstitial uromodulin regulates macrophage number and function in the kidney [46,79]. Hence, the maladaptive role of high uromodulin production in the transplant setting could be linked to its immunomodulatory properties in the renal interstitium. Uromodulin can enhance the expression of MHC II in macrophages, thereby increasing their potential to present antigens and contributing to an immunostimulatory environment within the kidneys. This ongoing state of immune stimulation in patients with high uromodulin production may negatively impact graft function, partially explaining the heightened risk of long-term graft failure associated with elevated uromodulin levels. Additionally, it is worth noting that when uromodulin was evaluated post-kidney transplantation, a bimodal association was identified, where the lowest and highest tertiles were protective, but the middle tertile had a higher risk of fibrosis and tubular atrophy [302,303]. A bimodal pattern has also been observed for the association between urinary uromodulin and renal morphology; more severe IFTA was noted in the middle tertile of uromodulin excretion. The lower values of uromodulin excretion (1st tertile) corresponded to cases without significant kidney damage and favorable outcomes. Higher values of uromodulin excretion (3rd tertile) would indicate cases where uromodulin upregulation has been successful, leading to favorable outcomes. The average values of uromodulin excretion (2nd tertile) would represent cases where uromodulin failed to upregulate (or where the upregulation mechanism has been exhausted due to prolonged damage triggers), leading to a progressive decline in kidney function, possibly culminating in graft loss. Urinary uromodulin is not merely a reflection of the total functioning mass of Henle’s loop but rather a target of inducible regulation and an active participant in the intricate processes of kidney disease pathogenesis [303]. The heightened excretion of uromodulin could potentially be attributed to increased synthesis in the kidney, increased shedding into the urine, or possibly both mechanisms at play [38,304]. Other studies have indicated improved outcomes with reduced graft failure when higher levels of serum uromodulin were present post-transplantation [92,287].

### 5.11. Fanconi Syndrome

The renal Fanconi syndrome is distinguished by glucosuria with normal serum glucose levels, generalized hyperaminoaciduria, urinary loss of bicarbonate, lactate, phosphate, potassium, and low-molecular-weight proteins [305]. These features can exhibit significant variability, and a considerable number of patients may present with only a subset of these characteristics. Nonetheless, the loss of low-molecular-weight proteins typically remains consistent. In children with Fanconi syndrome, the key symptoms and signs include polyuria, metabolic acidosis, rickets, and severe growth failure if not promptly treated [306].

Using capillary electrophoresis mass spectrometry, urinary proteome analysis of pediatric patients with Fanconi syndrome showed a reduced number of fragments derived from osteopontin and uromodulin, indicating loss of function of tubular excretion due to a disturbance of the endoplasmic reticulum in all patients with Fanconi syndrome, regardless of the underlying cause. Furthermore, there were alterations in the levels of six distinct fragments of the collagen alpha-1 (I) chain, with some showing elevated levels, whereas others were reduced in the urine. This suggests a shift in proteases involved in collagen degradation, which is often observed in cases of interstitial fibrosis. These changes were evident regardless of the stage of Fanconi syndrome, indicating that fibrosis might be an early underlying cause leading to the development of kidney insufficiency in patients with Fanconi syndrome [305].

### 5.12. Fabry Disease

Fabry disease is an X-linked disorder that follows gonosomal recessive inheritance. It is caused by a deficiency of alpha-galactosidase A. Due to this enzyme defect, there is a substantial buildup and storage of the undegraded substrate globotriaosylceramide (Gb3) in the lysosomes of various cell types [307]. In a study of 15 male Fabry disease patients at various clinical stages of the disease, significant quantitative and qualitative alterations in urinary uromodulin excretion were observed, suggesting a gradual decline in urinary uromodulin output. In certain cases, this decrease was accompanied by aberrant proteolytic processing of uromodulin. The variability in uromodulin excretion appeared to largely correspond to the severity of the phenotypic presentation in most patients [308]. Urinary proteome analysis of Fabry treatment-naive patients revealed an increased expression of uromodulin, which could potentially play a role in kidney damage at the tubular level, as well as contribute to inflammation and immune responses within the kidney [309,310]. In all patients participating in enzyme replacement therapy, the urinary uromodulin excretion patterns were normalized. Patients receiving substrate reduction therapy also experienced some degree of normalization in urinary uromodulin excretion, although to a lesser extent. Enzyme replacement therapy resulted in the normalization of urinary uromodulin excretion patterns in all patients. Substrate reduction therapy also led to some level of normalization in urinary uromodulin excretion, although it was not as comprehensive as that observed with enzyme replacement therapy [308].

## 6. Conclusions

Uromodulin is a pleiotropic and overall protective protein, leading us to expect compensatory upregulation upon kidney damage to fulfill its protective role. Therefore, it is suggested that in the case of kidney damage, uromodulin is upregulated and contributes to the repair and regeneration processes. However, in conditions where persistent kidney injury occurs, the initial compensatory upregulation of uromodulin is followed by a state in which its excretion reaches a fixed intermediate level, likely due to the failure of further upregulation. If this is the case, then different groups of uromodulin excretion levels represent distinct scenarios. 

Numerous studies have investigated the role of both serum and urinary uromodulin as potential biomarkers. An increasing body of evidence indicates that elevated levels of uromodulin, whether in the urine or serum, are independently associated with a reduced risk of developing acute and chronic kidney disease, as well as the progression of kidney disease, cardiovascular disease, and mortality. There appears to be an inverse correlation between serum uromodulin levels and the markers of systemic inflammation. However, conflicting findings have been reported. Some studies have proposed that urinary uromodulin may be associated with an increased risk of hypertension and CKD. 

The diverse functions of uromodulin and its interactions within the kidney highlight the complexity of kidney disease pathology. Targeted therapies can be tailored to each patient’s unique needs by identifying specific uromodulin-related molecular pathways and their impact on disease progression. Genetic studies have already shown that variations in the *UMOD* gene can influence uromodulin production and function, potentially affecting susceptibility to kidney diseases. Integrating genetic data with uromodulin biomarker levels could enable the identification of individuals at high risk of kidney diseases or those who may respond better to certain treatments. Our understanding of the biology and functions of uromodulin remains incomplete, warranting further research to unravel its complex implications for various health conditions. Continued research efforts will undoubtedly pave the way for improved diagnostic tools and innovative treatments for patients with kidney diseases.

## Data Availability

Not applicable.
